# The Transmembrane Envelope Protein of Porcine Endogenous Retroviruses Modulates Cytokine Release and Gene Expression in Human Immune Cells

**DOI:** 10.3390/ijms27188219

**Published:** 2026-09-15

**Authors:** Jinzhao Ban, Andrea Schienke, Maike Quotschalla, Antje Brinkmann, Ludwig Krabben, Sebastian Rausch, Benedikt B. Kaufer, Reinhard Schwinzer, Fatih Noyan, Joachim Denner

**Affiliations:** 1 Institute of Virology, Free University Berlin, 14163 Berlin, Germany; jinzhao.ban@fu-berlin.de (J.B.); ludwig.krabben@gmail.com (L.K.); benedikt.kaufer@fu-berlin.de (B.B.K.); 2 Department of Gastroenterology, Hepatology, Infectious Diseases and Endocrinology, Hannover Medical School, 30625 Hannover, Germany; schienke.andrea@mh-hannover.de (A.S.); quotschalla.maike@mh-hannover.de (M.Q.); noyan.fatih@mh-hannover.de (F.N.); 3 Department of General, Visceral and Transplant Surgery, Hannover Medical School, 30625 Hannover, Germany; brinkmann.antje@mh-hannover.de (A.B.); schwinzer.reinhard@mh-hannover.de (R.S.); 4 Institute of Immunology, Free University Berlin, 14163 Berlin, Germany; sebastian.rausch@fu-berlin.de

**Keywords:** porcine endogenous retroviruses (PERV), transmembrane envelope protein p15E, immunosuppression, cytokines, cytotoxic cells

## Abstract

Porcine endogenous retroviruses (PERVs), their transmembrane envelope protein p15E and peptides corresponding to a domain in p15E that is highly conserved among retroviruses, the immunosuppressive (isu) domain, demonstrated immunosuppressive properties. They inhibited in vitro immune reactions, and induced release of IL-6 and IL-10 in human peripheral blood mononuclear cells (PBMCs). We recently showed that p15E of PERV expressed on 293T cells reduced MHC expression, induced cytokine release in co-incubated human PBMCs, and inhibited cytotoxic cells. The cell-surface expression of p15E was chosen to simulate an artificially introduced expression on a transplant. Here, a new construct with a higher expression of p15E and consequently higher effects on cytokine release was designed and used. An intracellular cytokine assay was newly developed and applied, whereas a commercial cytokine array was used to analyze the release of 105 cytokines into the supernatant. The differential gene expression was analyzed by sequencing the RNA of PBMCs incubated with p15E-expressing and wild-type 293T cells. PERV p15E induced an elevated expression of IL-10, IL-6 and 24 other cytokines and modulated the expression of hundreds of genes. Furthermore, coating porcine L23 cells with the synthetic isu peptide of the PERV p15E protein and subsequently co-incubating them with human PBMCs induced IL-10 production, whereas direct addition of the peptide to PBMCs alone did not induce cytokine production. These results demonstrate that the PERV p15E protein can modulate cytokine release by human immune cells and suppress their cytotoxic activity. This immunomodulatory property may have potential applications in protecting transplanted tissues from immune-mediated rejection.

## 1. Introduction

Retroviruses are immunosuppressive in the infected host, inducing immunodeficiencies or contributing to tumor progression and increased susceptibility to opportunistic infections, which may be fatal [1,2]. Immunodeficiency viruses such as human immunodeficiency virus type 1 (HIV-1) are well-characterized in this regard; however, gammaretroviruses, including murine and feline leukemia viruses (MuLV, FeLV), also exhibit immunosuppressive properties. Endogenous retroviruses likewise play immunomodulatory roles. In the placenta, the envelope proteins of endogenous retroviruses, known as syncytins, have been co-opted, “enslaved”, to mediate the formation of the syncytiotrophoblast in humans and other species and to support immunoprotection of the embryo [3,4]. In tumors, envelope proteins derived from endogenous retroviruses may similarly contribute to tumor progression [5,6,7].

There is compelling evidence that the transmembrane (TM) envelope proteins are responsible for retrovirus-induced immunosuppression [8,9]. Within these TM proteins, a region highly conserved across retroviruses, termed the immunosuppressive (isu) domain, has been identified. Recombinant and purified viral proteins, as well as synthetic peptides corresponding to the isu domain, have been shown to inhibit immune responses both in vitro and in vivo, to modulate cytokine production and gene expression [2,8,9,10,11,12,13,14,15,16]. Notably, single mutations within the isu domain abolish its immunosuppressive activity [17,18].

Importantly, there is strong evidence that TM envelope proteins and their isu domains are functionally active in vivo, as demonstrated in immunization studies [19,20,21] and tumor rejection models [18,22,23,24,25]. In the latter, certain mouse tumor cells form tumors in severe combined immunodeficiency (SCID) or irradiated mice, but were rejected in immunocompetent mice. However, expression of retroviral TM envelope proteins or even the isu domain alone enables these tumor cells to grow in immunocompetent hosts. This finding indicates that TM envelop proteins can suppress host immune responses, thereby preventing tumor rejection.

Inactivated virus preparations, the TM envelope protein p15E, and the isu peptide of porcine endogenous retroviruses (PERVs) have also been shown to exert immunosuppressive effects [11,26,27]. PERVs became a major focus in the development of xenotransplantation, a technology that utilizes cells, tissues, and organs from genetically modified pigs to address the shortage of human donor organs and to treat diabetes [28,29]. PERV-A and PERV-B are integrated into the genome of all pigs, whereas PERV-C, the evolutionarily youngest subtype, is present in most but not all pigs [30]. In some animals, recombination events between PERV-A and PERV-C have been observed, generating PERV-A/C recombinants characterized by higher replication rates and increases in the number of repeat sequences in the long terminal repeats (LTRs) of the virus, representing transcription factor binding sites [31,32]. Under certain conditions, PERV-A, PERV-B, and PERV-A/C can produce infectious viral particles capable of infecting human cells in vitro [31,33]. However, except in minipigs, the in vivo expression of PERV proteins is generally very low [34,35].

Based on findings that the TM envelope proteins of different retroviruses protect tumor cells from rejection in immunocompetent mice [22,23,24,25], the concept emerged to use a retroviral TM envelope protein to protect xenotransplants from immune rejection [36,37]. Among these proteins, the TM envelope protein p15E of PERV appears particularly suitable because, as a protein encoded by an endogenous retrovirus, it does not elicit antibody production in pigs, which are immunologically tolerant to it [38].

In an initial proof-of-principle study, cells expressing PERV p15E were shown to modulate cytokine expression in co-incubated human peripheral blood mononuclear cells (PBMCs), including increased expression of IL-10 and IL-6 [39]. In addition, expression of p15E downregulated MHC in the transfected cells and reduced the activity of cytotoxic cells. In the present study, a different construct enabling substantially higher p15E expression was generated, and the effects of p15E on cytokine production and gene expression were investigated in greater detail using additional experimental approaches.

## 2. Results

### 2.1. Generation of p15E Expressing 293T Cells

**Figure 1:**
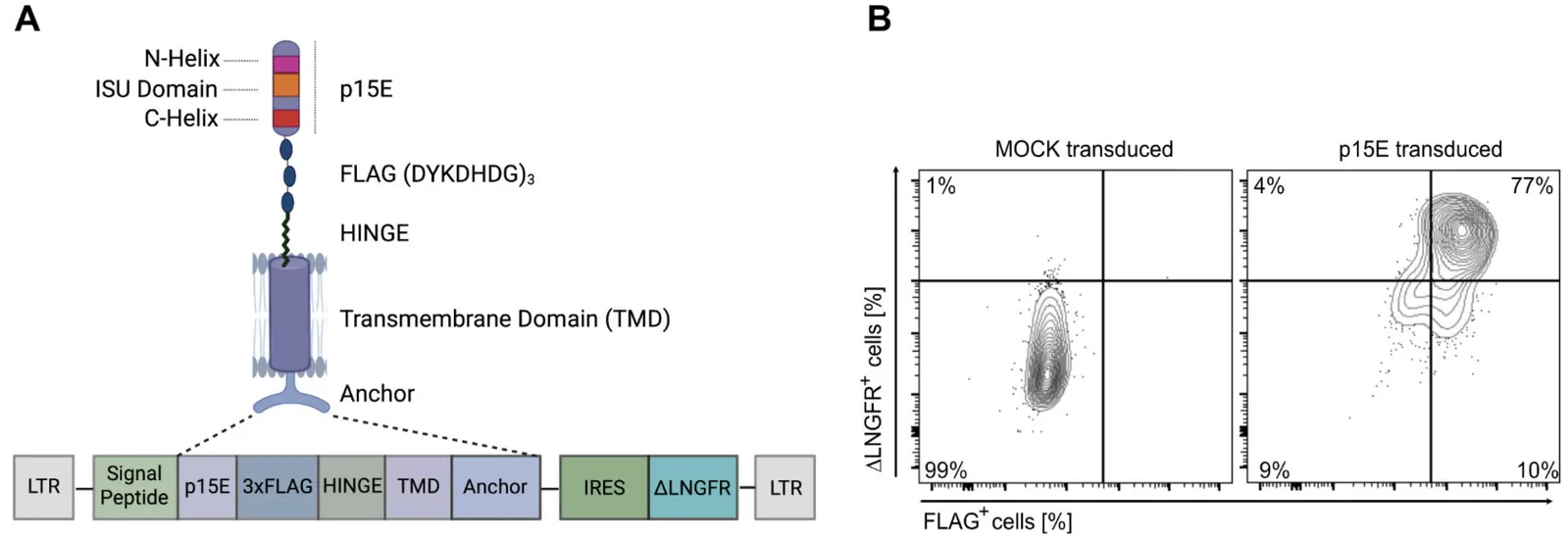
Schematic representation and flow cytometric validation of the γ-retroviral p15E expression construct. ((**A**), upper panel) Structural diagram of the p15E surface expression module. The extracellular portion consists of the p15E sequence of PERV, encompassing the N-terminal helix, the C-terminal helix, and the immunosuppressive (isu) domain located proximal to the Cys-Cys loop; one of the three cysteines within this region was mutated to serine to prevent intermolecular disulfide bond formation. A triple FLAG epitope tag (DYKDHDG-DYKDHDI-DYKDDDDK) is fused C-terminally to p15E to enable antibody-based detection. A hinge region connects the ectodomain to the transmembrane domain (TMD) and intracellular anchor sequence of MHC class I, which mediate stable surface tethering in the correct orientation. ((**A**), lower panel) Linear map of the retroviral expression cassette flanked by long terminal repeats (LTRs). ΔLNGFR (CD271) is co-expressed via an IRES and serves as a surface-accessible reporter gene. (**B**) Representative dot plots showing surface co-expression of FLAG-tagged p15E and ΔLNGFR in mock- and p15E-transduced 293T cells as assessed by flow cytometry. 77% of p15E-transduced cells were double positive, confirming efficient surface expression of the construct.

To achieve stable surface expression of the p15E transmembrane protein of PERV, an expression construct was designed following the membrane-anchoring strategy previously established for membrane-bound IL-2 (mbIL-2) [40]. The bicistronic retroviral cassette encodes the extracellular part of p15E—containing the N-terminal helix, C-terminal helix, and isu domain, with one cysteine mutated to prevent intermolecular disulfide bonds—fused to a triple FLAG tag, a hinge, and an MHC class I transmembrane anchor for native surface localization. The signal peptide of the ENV precursor protein of gp70 and p15E was positioned in front of the construct to establish extracellular plasma membrane location. A downstream IRES drives co-expression of low-affinity nerve growth factor receptor (ΔLNGFR; CD271), which served as a transduction marker (Figure 1). Human embryonic kidney (HEK) 293T cells were transduced with γ-retroviral particles encoding this construct, resulting in stable genomic integration and stable p15E surface expression. Flow cytometric analysis of transduced cells revealed co-expression of FLAG-tagged p15E and ΔLNGFR in 77% of cells, confirming efficient transduction and surface display of the construct, whereas mock-transduced cells remained negative for both markers (Figure 1B).

### 2.2. Analysis of the Expression of p15E

**Figure 2:**
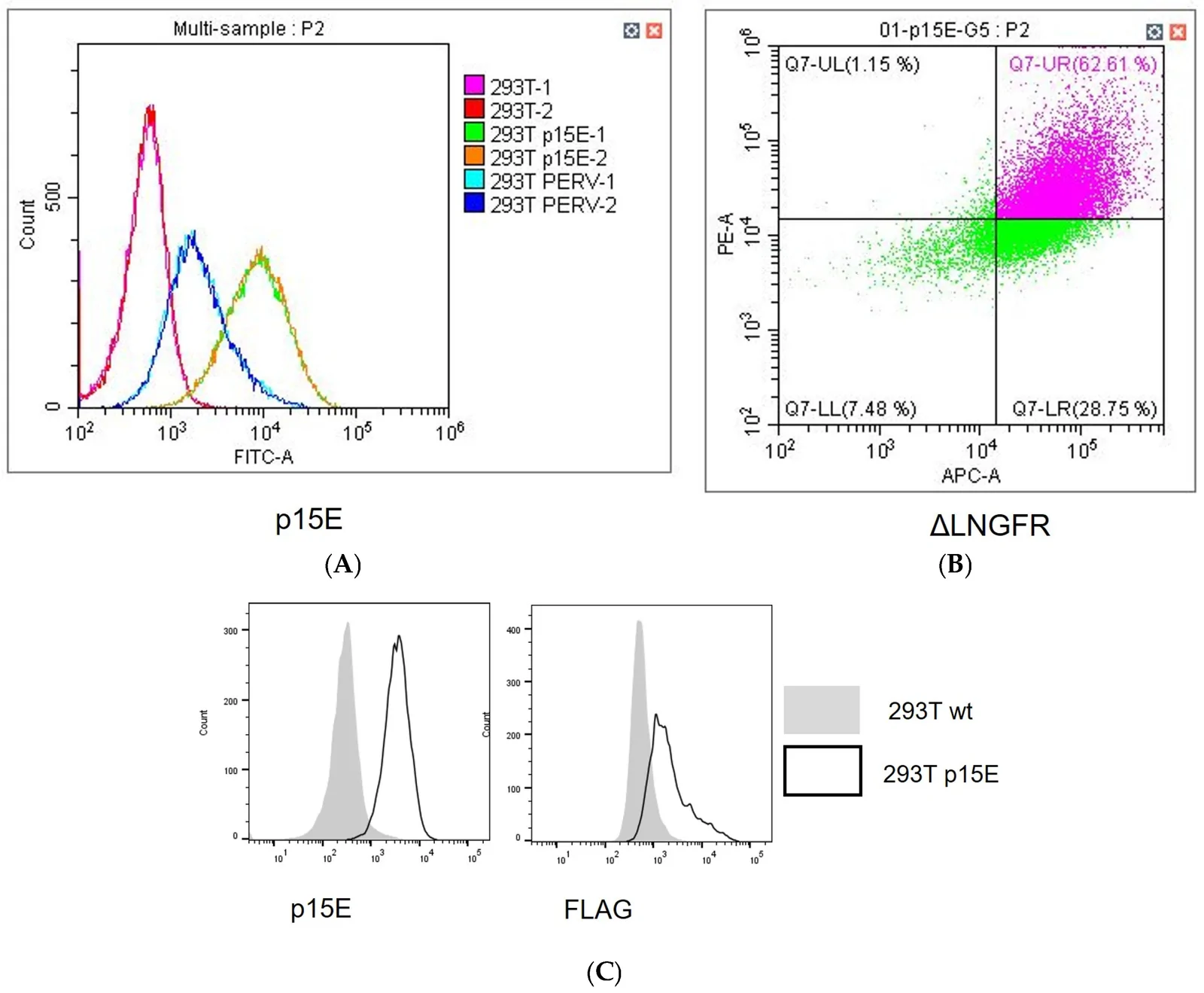
FACS analysis of the expression of p15E, ΔLNGFR and FLAG in wt 293T cells, in 293T cells expressing p15E and in 293T cells producing PERV-A/C in two institutes. (**A**), two different batches of each cell type were tested using a goat serum against p15E of PERV (serum #355) and a FITC-marked anti-goat antiserum (performed at the Institute of Virology, Berlin). (**B**), flow cytometry dot plot showing the expression of FLAG using a PE-labeled rat anti-FLAG antibody and of ΔLNGFR using an APC-labeled mouse anti-human CD271 antibody on p15E-expressing 293T cells (performed at the Institute of Virology, Berlin). (**C**), results of the FACS analysis of 293T cells expressing p15E and wt 293T cells using goat serum against p15E of PERV (serum #355) and a different serum against FLAG (see Section 4.6) (performed at the Medical School Hannover).

Expression of the construct was analyzed using antibodies against p15E, FLAG, and ΔLNGFR (Figure 2). In addition to wild-type (wt) 293T cells and 293T cells expressing p15E, 293T cells infected with and producing PERV-A/C were also examined. This virus was originally isolated from pig PBMCs [41] and subsequently passaged several times on human 293T cells, resulting in increased replication rates and expansion of repeat sequences within the LTR of the integrated virus [31]. These LTR sequences were shown to change over time [42]. The virus used in the present experiments possessed an LTR length intermediate between those of PERV-3° and PERV-5° and therefore represents a highly replicating variant [39].

Surface expression of p15E, detected using a specific antiserum against p15E [43], was observed both in 293T cells expressing the p15E construct and in PERV-producing cells (Figure 2A). Although the p15E construct exhibited a stronger signal than the PERV-producing cells, a direct comparison of expression levels is difficult because the proteins are not identical and may adopt different conformations, potentially affecting the binding affinity of the primary antibody. The expression of the p15E construct was verified using antibodies against FLAG and ΔLNGFR (Figure 2B,C), measuring 63.76% FLAG-positive cells and 91.36% ΔLNGFR-positive cells (Figure 2B).

### 2.3. Influence of p15E on IL-6 and IL-10 Release

**Figure 3:**
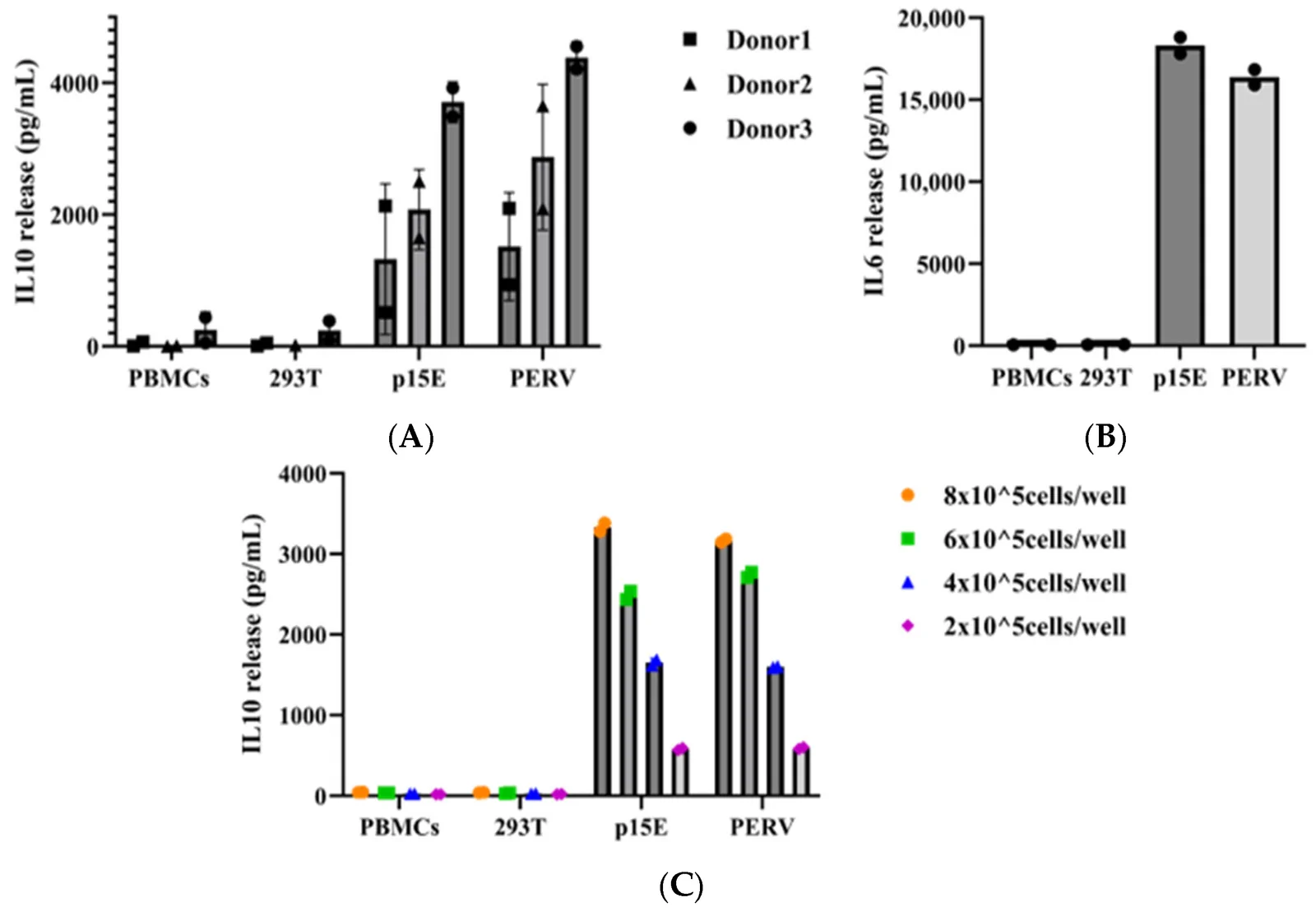
Increase in IL-10 and IL-6 expression after co-cultivation of 293T cells expressing p15E or producing PERV in comparison to controls. (**A**), IL-10 was tested in the supernatant of PBMCs alone, PBMCs plus wt 293T cells, PBMCs plus 293T cells expressing p15E (p15E) and PBMCs plus 293T cells producing PERV (PERV), using PBMCs from three different donors. Data is presented as mean ± SD. Each dot represents an independent experiment, and the bars indicate the mean values. (**B**), the same as in A with Il-6 with PBMCs from one donor, two samples from one donor were tested, the columns mark the medium. (**C**), Equal amounts of cells (1.5 × 10^4^ cells/well), either wt 293T, 293T expressing p15E, or 293T producing PERV, were incubated with different amounts of PBMCs. Two samples from one donor were tested, the columns mark the medium.

When 293T cells expressing p15E were co-cultured with human PBMCs, release of IL-6 and IL-10 into the culture supernatant was significantly increased compared with PBMCs co-incubated with control wt 293T cells as observed by ELISA (Figure 3A,B). The cytokine amount in the supernatant was measured after 24 h since previous results using the isu peptide of HIV-1 indicated a peak of IL-10 and IL-6 release between 16 to 24 h [10]. As previously reported for the isu peptide of HIV-1 [10], PBMCs from different donors exhibited variable levels of IL-10 production despite exposure to equal numbers of p15E-expressing cells (Figure 3A). Whereas co-cultivation of 293T cells expressing the previously described p15E constructs with human PBMCs resulted in a maximum IL-10 release of 500 pg/mL [39], co-cultivation with the p15E construct used in the present study resulted in a substantially higher IL-10 release of 3500 pg/mL (Figure 3).

Increasing the number of human PBMCs while maintaining a constant number of p15E-expressing 293T cells resulted in a corresponding increase in IL-10 production, indicating that p15E-expressing 293T cells were able to stimulate at least the highest number of PBMCs tested to secrete IL-10 (Figure 3C).

These experiments also show that PBMCs alone and PBMCs co-incubated with wt 293T do not release significant amounts of IL-10 or IL-6.

### 2.4. Generation of a New Cytokine Assay Based on Flow Cytometry

In addition to ELISA-based measurements of cytokine release into the culture supernatant, an assay was established to analyze intracellular cytokine expression. In this approach, cytokine secretion was blocked with Brefeldin A, allowing intracellular accumulation of cytokine proteins. Brefeldin A is a guanine nucleotide exchange factor, it inhibits vesicle formation and transport between the endoplasmic reticulum and the Golgi apparatus. Cytokine-specific antibodies were used to quantify cytokine-positive cells by flow cytometry. In parallel, cell surface markers were analyzed to identify and characterize T cells, B cells, and monocytes.

**Figure 4:**
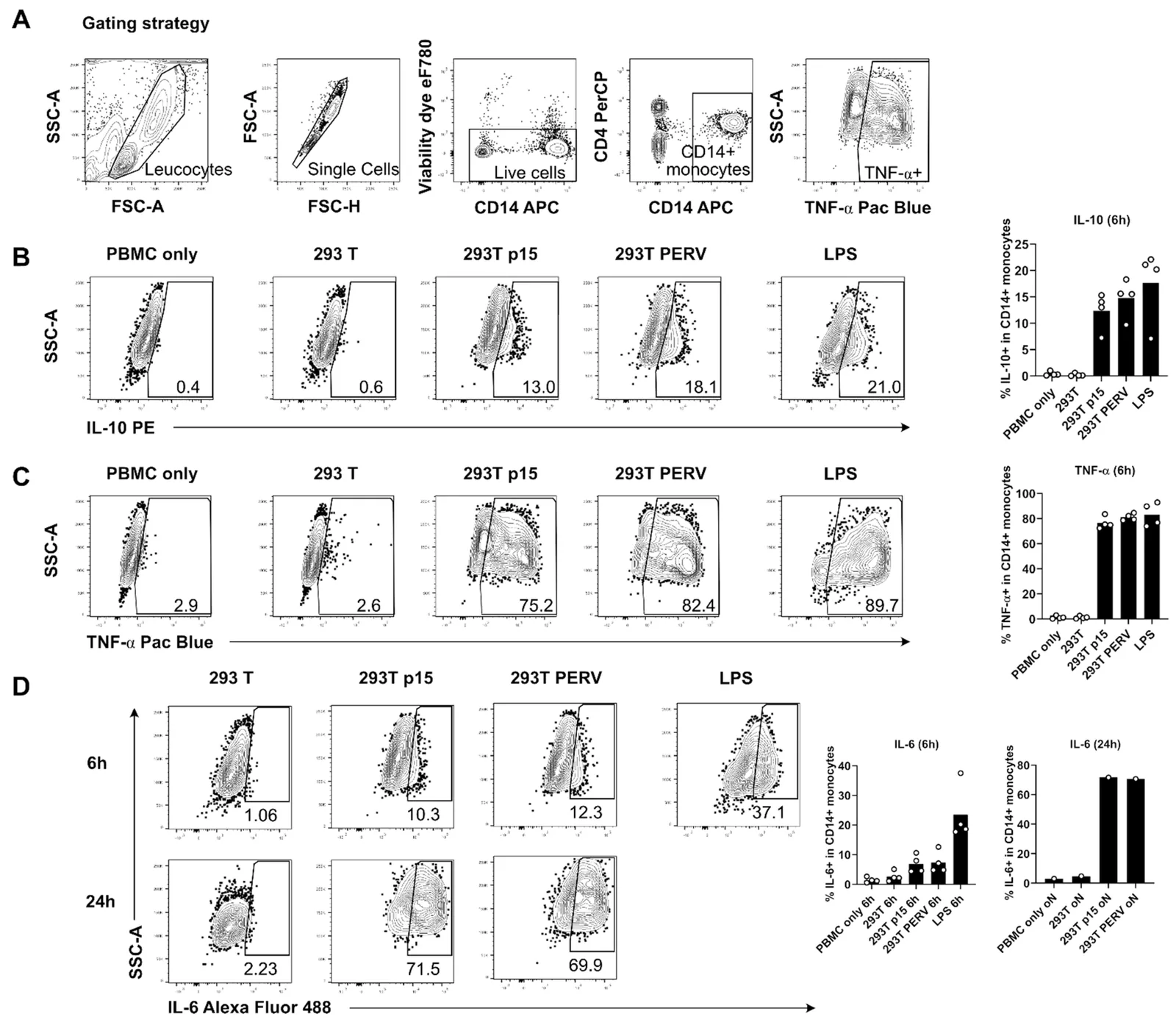
Intracellular detection of cytokine production by CD14+ monocytes. Human PBMC were co-cultured with wt 293T, 293T p15E, or PERV-producing 293T followed by surface staining and intracellular detection of cytokine production. (**A**), Gating strategy. (**B**–**D**), Representative FACS plots depicting the production of IL-10 (**B**), TNF-α (**C**), and IL-6 (**D**) by live CD14+ monocytes after 6 h of culture with wt 293T, 293T p15E, or 293T PERV-producing cells. Plots in the top row of (**D**) represent cultures stimulated for 6h, plots in the lower row represent overnight cultures. The data were derived from two independent experiments performed with PBMC from one donor in one experiment and from three donors in the second experiment (circles), the columns show the mean.

To optimize the incubation period, several protocols were tested: (i) 24 h co-incubation plus five hours in the presence of Brefeldin A, (ii) overnight co-incubation followed by five hours with Brefeldin A (total incubation time of 24 h), and (iii) a six hours incubation including the final five hours with Brefeldin A. Both the six hours and 24 h incubation protocols (including 5 h Brefeldin A) produced optimal results (Figure 4, Table 1) and were therefore selected for all subsequent experiments.

IL-10 and TNF-α production was readily detectable in CD14^+^ monocytes following six hours of co-culture with 293T expressing p15E and with PERV-producing 293T cells, but not in monocytes cultured with 293T wild type cells (Figure 4B,C). Monocytes cultured with 293T cells expressing p15E or PERV producing 293T cells produced little IL-6, however, robust IL-6 production was seen following overnight incubation with 293T p15E and PERV-producing cells (Figure 4D). Next to monocytes, CD21^+^ B cells responded with significant TNF-α and modest IL-6 production in co-culture with p15E-expressing and PERV-producing 293T cells, but not in cultures kept with wt 293T cells or when stimulated with LPS. IL-10 was not detectable in B cells. Finally, T cells identified by CD3 and CD4 expression did not produce any of the surveyed cytokines under the tested conditions.

It is important to note that IL-6 and IL-10 production was low after the six hour incubation period, but increased markedly after 24 h (Figure 4B,D; Table 1). In contrast, TNF-α production was already high after 6 h and showed only a modest further increase after 24 h.

When the longest incubation protocol (24 h followed by 5 h in the presence of Brefeldin A) was analyzed, only very low intracellular levels of IL-10, IL-6, and TNF-α were detected (Table S1; Figure S1), indicating that by the 24 h time point a substantial proportion of these cytokines had already been secreted into the culture medium before Brefeldin A was added.

### 2.5. Increase in Cytokine Expression Detected by Real-Time RT-PCR

**Figure 5:**
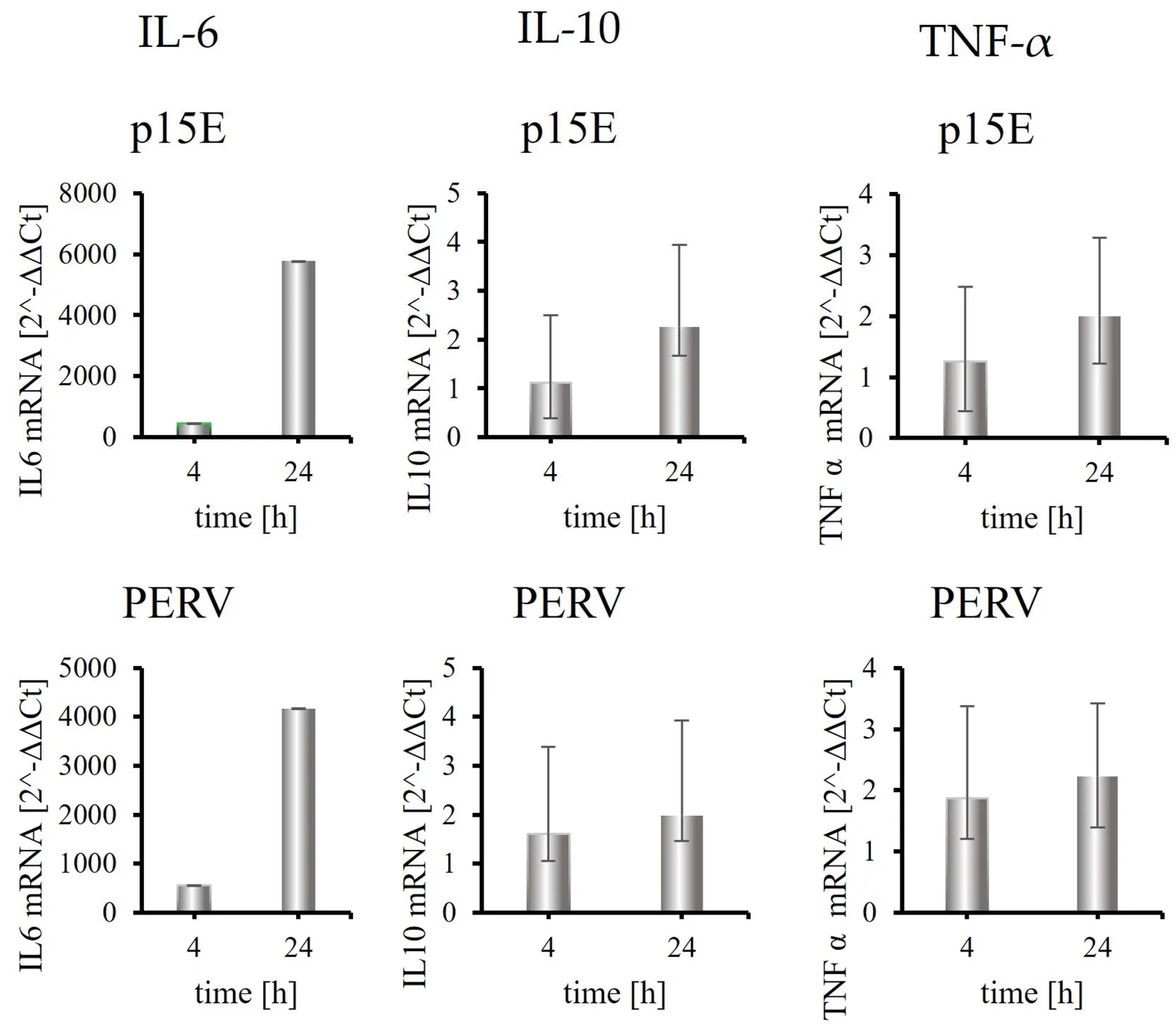
Changes in the mRNA expression of IL-6, IL-10 and TNF-α in human PBMC co-cultivated with 293T cells expressing p15E (p15E) and or 293T cells producing PERV (PERV). Please note that the 2^−ΔΔCt^ value represents the relative change in cytokine expression normalized to GAPDH expression.

A real-time RT-PCR analysis was performed to assess changes in the expression of cytokines, including IL-10 and IL-6 (Figure 5). Determination of expression levels at multiple time points, as previously conducted for the isu peptide of HIV-1 [10] and the recombinant TM envelope protein of HERV-K [11], was not feasible due to differences in the experimental setup. In those earlier studies, the peptide or TM envelope protein were added directly to human PBMCs, and RNA was isolated after incubation. In the present study, p15E-expressing 293T cells were co-cultured with human PBMCs. Because the two cell populations could not be separated, total RNA was isolated from both cell types and used for real-time RT-PCR analysis, with the understanding that 293T cells do not express cytokines as shown here (Figure 3 and Figure 4) and previously [39]. After 24 h, a clear increase in the expression of all analyzed cytokines was observed (Figure 5). Notably, the actual level of cytokine expression may be underestimated, as the 293T cells underwent approximately one round of division during this period [44], leading to additional RNA that diluted the PBMC-derived signal. An increase in mRNA expression was also observed for MMP-1 and TNF-α, but not for IFN-γ.

### 2.6. Modulation of Cytokine Expression Detected by a Cytokine Array

A membrane-based cytokine array was performed which analyzes the expression of 105 proteins. 24 cytokines were found to be increased in expression when 293T cells ex-pressing p15E were co-cultivated with human PBMCs in comparison to PBMCs co-cultivated with wt 293T cells (Table 2).

The most up-regulated cytokines were CCL3/CCL4 (MIP-1α/MIP-1β), CCL20 (MIP-3α), CXCL1 (GROα), IL-1β, and RANTES. Similar results had been observed in previous experiments analyzing the influence of the isu peptide of HIV-1 [10] and the transmembrane envelope protein of HERV-K produced in yeasts [11] on the cytokine release of human donor PBMCs (Table 2).

### 2.7. Effect of p15E on Cytotoxicity of Human Natural Killer (NK) Cells

**Figure 6:**
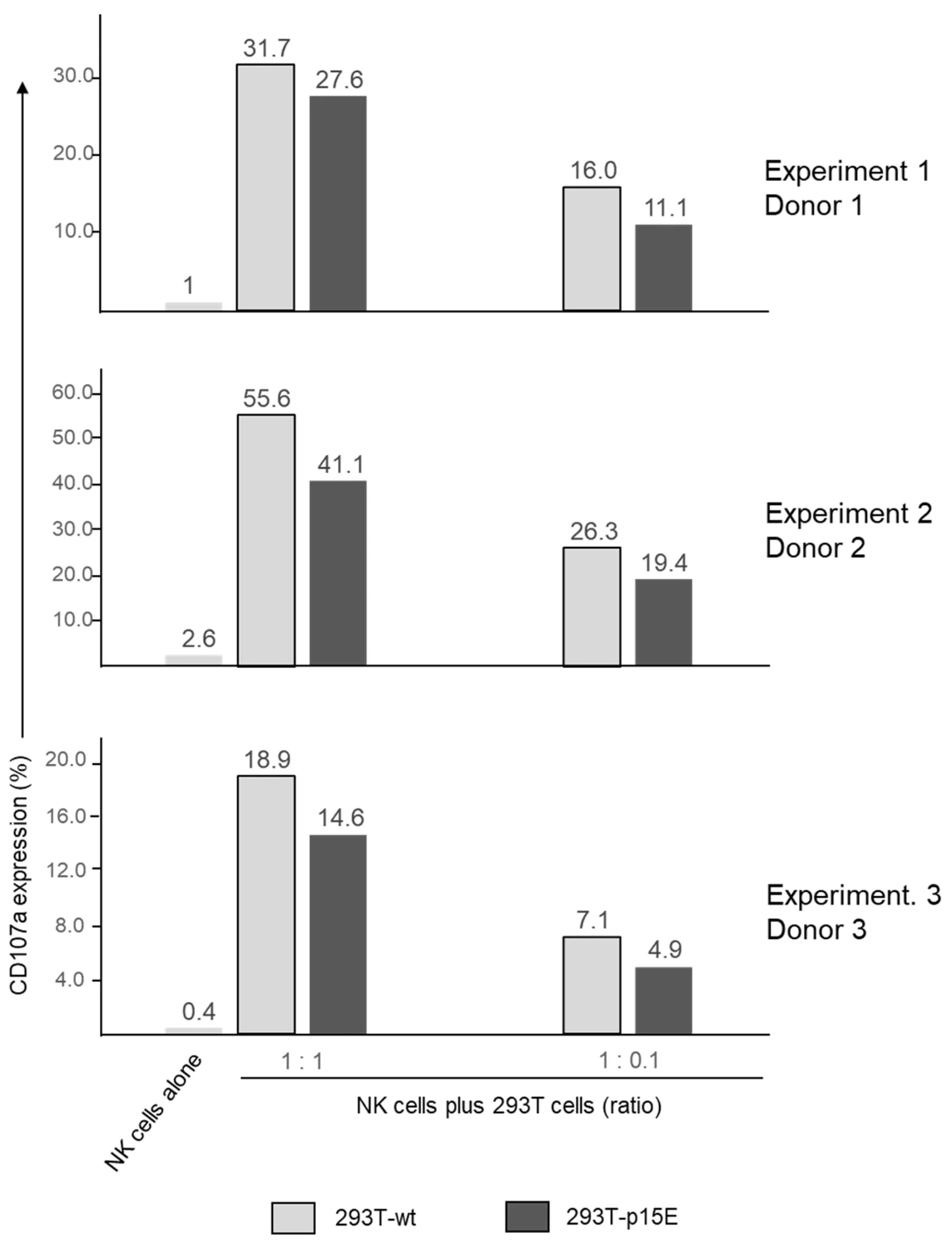
Degranulation of NK cells from three different donors by co-culturing with 293T cells in three independent experiments. NK cell-enriched effector populations were obtained by depletion of CD4^+^CD8^+^HLA-DR^+^CD14^+^ cells from PBMCs. The cells were cultivated alone, with wt 293T cells (grey columns) or 293T cells expressing p15E (dark columns). Expression of CD107a was monitored after 2 h on gated CD56^+^CD45^+^ cells.

Human NK cells (CD56^+^CD3^−^) were enriched from PBMCs by depletion of CD4^+^CD8^+^HLA-DR^+^CD14^+^ cells using appropriate antibodies and magnetic cell sorting (MACS). The cells were cultivated for 2 h alone or in co-culture with wt 293T and 293T cells expressing p15E. NK cells alone did not express significant levels of CD107a (0.4 to 2.6%) (Figure 6). Using NK cells from three blood donors and applying two different NK:293T ratios, stronger CD107a expression on NK cells were found after contact with wt 293T cells compared to 293T cells expressing p15E cells. A statistically significant difference was observed between NK cells co-cultured with 293T cells (1:0.1) and NK cells co-cultured with p15E-expressing 293T cells (1:0.1), with a *p*-value < 0.05. For the NK + 293T (1:1) and NK + p15E (1:1) groups, the paired *t*-test yielded a *p*-value of 0.0516. These patterns suggest that degranulation/cytotoxic activity of NK cells is reduced when target cells express p15E.

### 2.8. Influence of p15E on Gene Expression

Three batches of 293T cells expressing p15E and three batches of wt 293T cells were incubated with human donor PBMCs for 24 h, the RNA was isolated from all six batches. Since wt 293T cells and PBMCs could not be divided the isolated RNA corresponds to both cell types. All six RNA samples were sequenced and an analysis was performed, which genes were up-regulated and which were down-regulated in the PBMCs co-incubated with 293T cells expressing p15E in comparison to the PBMCs co-incubated with the wt 293T cells (Table 3 and Table 4).

**Figure 7:**
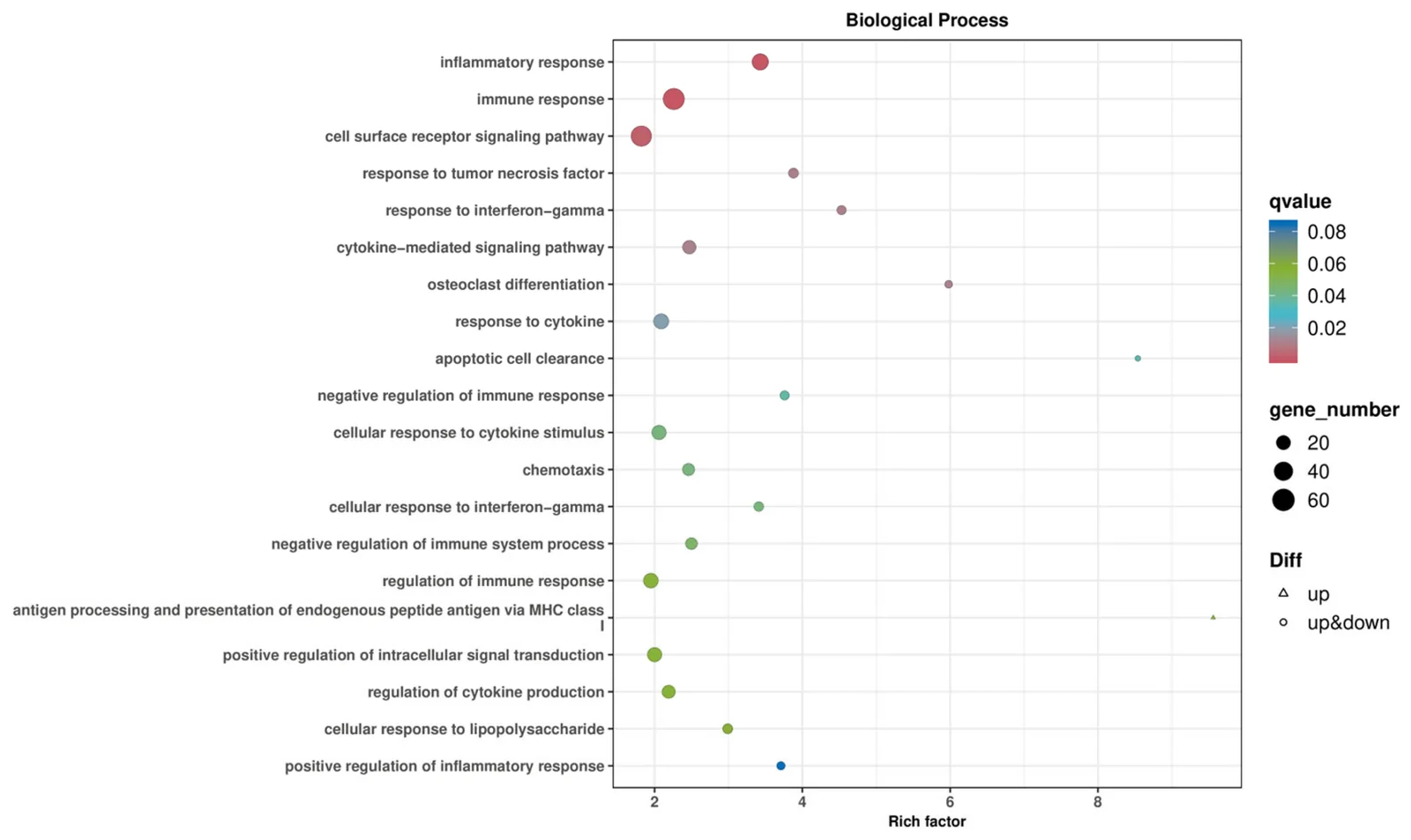
Presentation of the changes in functional annotation (GO Analysis, Gene Ontology) comparing the gene expression in human PBMCs co-incubated with 293T cells expressing PERV p15E with the gene expression in human PBMCs co-incubated with wild-type 293T cells.

**Figure 8:**
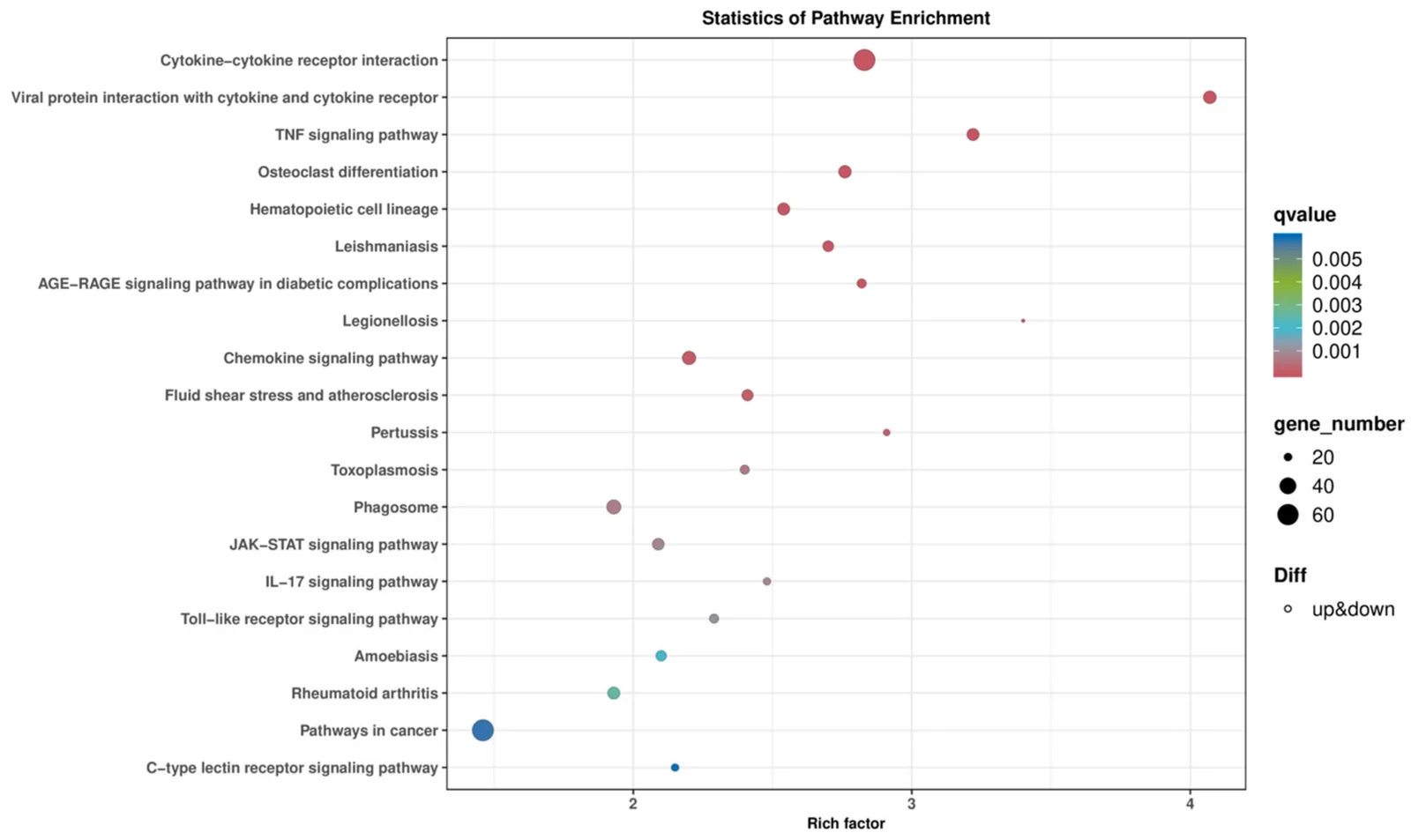
Results of the functional enrichment analysis (KEGG Pathways analysis, Kyoto Encyclopedia of Genes and Genomes) comparing the gene expression in human PBMCs co-incubated with 293T cells expressing PERV p15E with the gene expression in human PBMCs co-incubated with wild-type 293T cells.

A total of 14,882 genes were analyzed in the RNA sequencing dataset. The correlation plot, the MA plot and the volcano plot are shown in Figure S2. A final set of differentially expressed genes (DEGs) was obtained by applying statistical significance filtering (FDR < 0.05) together with a fold-change cutoff (|log2FC| > 1), yielding a curated gene list of 1736 genes, 800 genes were up-regulated, 936 genes were down-regulated. Gene Ontology (GO) enrichment analysis revealed that differentially expressed genes were predominantly associated with inflammatory response, immune response, and cell surface receptor signaling pathways (Figure 7). The Kyoto Encyclopedia of Genes and Genomes (KEGG) enrichment dot plot showed that cytokine–cytokine receptor interaction was the most significantly enriched pathway with a high gene count, indicating a strong involvement of immune signaling (Figure 8).

When the up- and down-regulated genes were compared with the results of sequencing analysis of PBMCs treated with the HIV isu peptide homopolymer [10] or the recombinant TM envelope protein of HERV-K produced in yeasts [11], the same genes were up- and down-regulated (Table 3 and Table 4). Please note the differences in the presentation of the results. Whereas in the previous analysis 50 up- and 50 down-regulated were presented, here immune system-associated genes were picked, their position in the list of all up- and down-regulated genes was indicated as well as the position of the corresponding gene in the studies with HIV and HERV-K. In all three studies, MMP-1, IL-6, CXCL1, TREM1, IL-1β and CXCL3 were the genes with the highest up-regulation (Table 3) and TREM2, FCN1, and CD36 the genes with the highest down-regulation (Table 4). It is interesting that several HLA molecules were found down-regulated by p15E.

### 2.9. Influence of the Isu Peptide of PERV on Cytokine Release

In order to establish a system where the isu peptide of PERV is presented on pig cells, a synthetic peptide corresponding to the isu sequence of PERV was added to L23 cells. The L23 is a B cell subline of a lymphoblastoid cell line B1 from a dd haplotype miniature swine [45]. No reverse transcriptase activity was found in the supernatant of L23 cells, indicating that they did not release retroviruses [45]. PERV-A, PERV-B, PERV-C and PERV-A/C were found by PCR in this cell line, the copy number of PERV pol was 55 as determined by droplet digital (dd)PCR [46]. However, the expression of PERV pol was very low (27% of the expression of mRNA in PK15 cells as measured by real-time RT-PCR), suggesting that no p15E protein was expressed [46].

**Figure 9:**
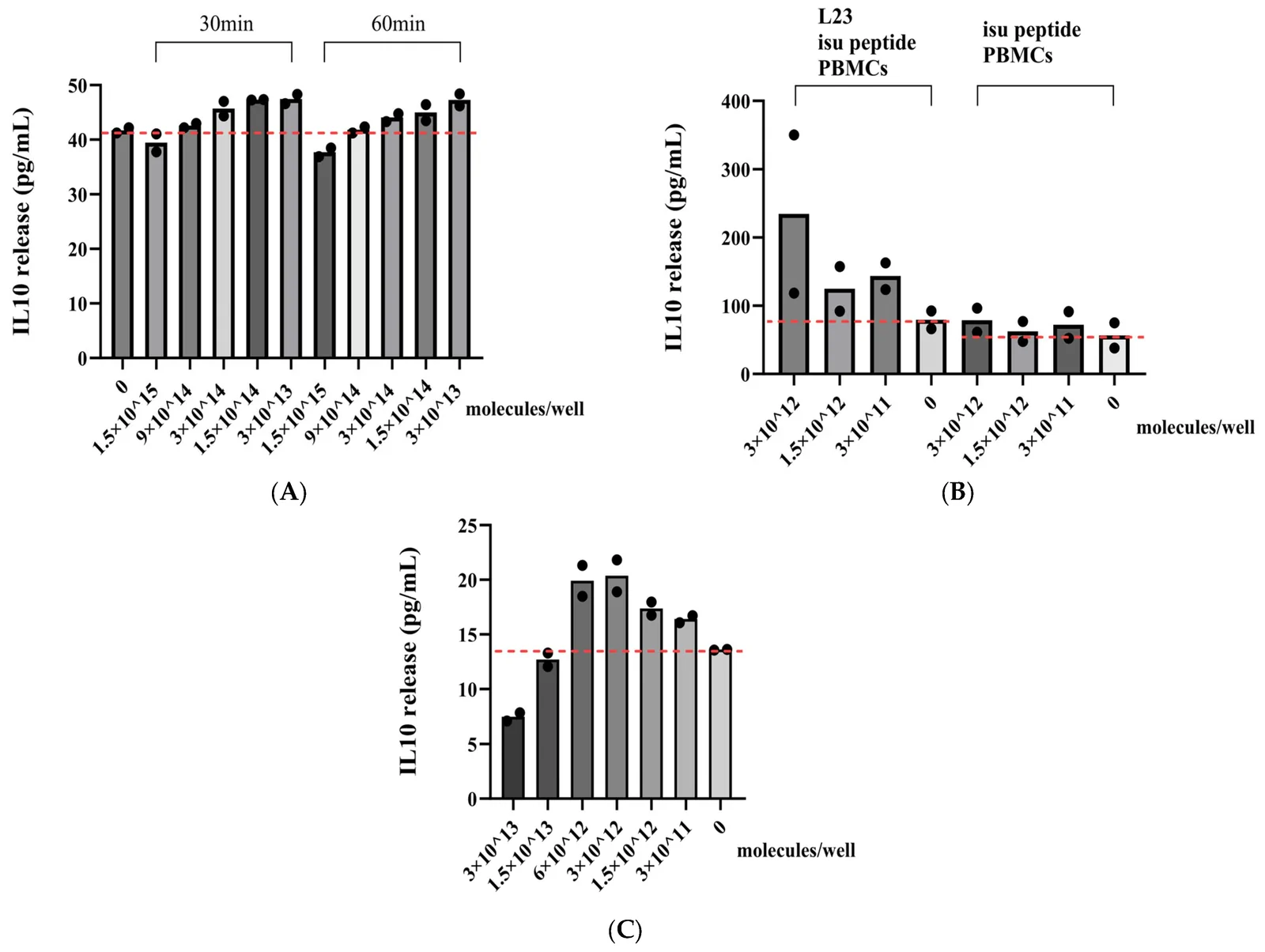
Effect of coating porcine L23 cells with the PERV isu peptide on IL-10 release by co-cultured human PBMCs. (**A**) Porcine L23 cells (3 × 10^5^ cells/well) were incubated with different amounts of the PERV isu peptide for 30 or 60 min and subsequently co-cultured with human PBMCs (3 × 10^5^ cells/well) for 24 h. IL-10 release in the co-culture supernatants was measured. The red line indicates the level without peptide. (**B**) Effect of coating porcine L23 cells with the PERV isu peptide on IL-10 release by co-cultured human PBMCs in comparison with isu peptide added directly to human PBMCs. Porcine L23 cells (1.5 × 10^5^ cells/well) were incubated with different amounts of the PERV isu peptide for 30 min and subsequently co-cultured with human PBMCs (3 × 10^5^ cells/well) for 24 h. For comparison, the peptide was added directly to the PBMCs. IL-10 release in the co-culture supernatants was measured. (**C**), Effect of coating porcine L23 cells with the PERV isu peptide on IL-10 release by co-cultured human PBMCs. Porcine L23 cells (1.5 × 10^5^ cells/well) were incubated with different amounts of the PERV isu peptide for 30 min and subsequently co-cultured with human PBMCs (3 × 10^5^ cells/well) for 24 h. IL-10 release in the co-culture supernatants was measured. The red line indicates the level without peptide.

When different concentrations of the peptide were incubated with L23 cells for 30 or 60 min before co-cultivation with human PBMCs, the highest peptide concentration resulted in reduced IL-10 release compared with the control incubation without peptide, whereas at lower peptide concentrations the IL-10 release increased (Figure 9A). When the number of L23 cells was reduced and lower amounts of peptide were used, a dose-dependent increase in IL-10 release was observed (Figure 9B). In the same experiment, addition of the peptide directly to human PBMCs in the absence of L23 cells did not induce IL-10 release. These findings confirm previous reports showing that isu peptides of gammaretroviruses [12,13] and HIV-1 [10,15] are inactive as monomers and become biologically active only when conjugated to bovine serum albumin (BSA) or presented as homopolymers.

In a separate experiment, a broader range of peptide concentrations was tested to determine the optimal amount for IL-10 induction. An intermediate peptide concentration induced the highest IL-10 release (Figure 9C), whereas both higher and lower peptide concentrations resulted in reduced IL-10 production.

In each experiment, PBMCs from a different donor were used (Figure 9). As expected, IL-10 production varied among donors, with some donors producing higher levels than others (see also Figure 3A and [10]).

## 3. Discussion

Evidence for the immunosuppressive properties of PERV p15E expressed on human 293T cells, previously reported in [39], was confirmed and further extended using several complementary approaches. A newly developed expression construct, designed to achieve higher levels of p15E surface expression than those used in earlier studies, produced more pronounced effects on peripheral blood mononuclear cells (PBMCs) from healthy human donors. The enhanced activity of this construct is likely attributable to two key modifications: incorporation of an MHC class I transmembrane anchor, which increased the stability of p15E at the cell surface, and retroviral transduction, which enabled stable genomic integration and sustained transgene expression. Robust surface expression of p15E was confirmed by flow cytometric analysis using specific antisera directed against p15E, the FLAG epitope tag, and the co-expressed ΔLNGFR reporter (Figure 2).

### 3.1. Influence of p15E on Cytokine Release, Relationship with Defensins

Consistent with the enhanced surface expression of p15E, co-culture of p15E-expressing cells with human PBMCs resulted in a marked increase in the production of IL-10 and IL-6, as demonstrated by ELISA (Figure 3) and PCR-based gene expression analyses (Figure 5). Among these, IL-10 is a key immunosuppressive cytokine known to inhibit cytokine secretion, reduce antigen-presenting cell function, and suppress CD4^+^ T-cell activation [47].

Both IL-10, a potent immunosuppressive and anti-inflammatory cytokine, and IL-6, a pleiotropic pro-inflammatory cytokine, were upregulated following exposure to p15E. This pattern is consistent with observations in vivo, where immunosuppressive retroviruses induce the simultaneous production of IL-10 and IL-6. Rather than simply suppressing immune responses, retroviruses promote a state of immune dysregulation characterized by concurrent activation of inflammatory and regulatory pathways. Consequently, the concurrent elevation of IL-6 and IL-10 is indicative of immune dysregulation rather than successful immune regulation. Similar alterations have been described in HIV-1 infection, where increased serum levels of IL-10 and IL-6, accompanied by elevated TNF-α and IFN-γ, are characteristic features of chronic infection [48,49,50,51].

To confirm the ELISA findings, an intracellular cytokine staining assay was established and applied for the first time to investigate p15E-induced cytokine modulation. The assay was based on inhibition of cytokine secretion by Brefeldin A, followed by flow cytometric analysis. Brefeldin A blocks protein transport from the endoplasmic reticulum to the Golgi apparatus, resulting in the intracellular accumulation of newly synthesized cytokines [52]. Simultaneous detection of intracellular cytokines, including IL-10, IL-6, and TNF-α, together with cell-surface markers such as CD14 (monocytes/macrophages), CD4 (T cells), and CD21 (B cells), enabled a detailed characterization of the effects of p15E on distinct PBMC subsets (Table 1, Figure S1). Comparable amounts of B cells were detected when CD21 was replaced by the alternative B-cell marker CD19. Whereas intracellular IL-10, Il-6, and TNF-α expression was higher after 24 h compared with 6 h of co-incubation, in a long term experiment (24 h followed by five hours in the presence of Brefeldin A) only very low intracellular levels of IL-10, IL-6, and TNF-α were detected (Table S1 and Table 1, Figure S1), indicating that by the 24 h time point a substantial proportion of these cytokines had already been secreted into the culture medium before Brefeldin A was added.

These findings are consistent with previous studies investigating the kinetics of IL-10 and IL-6 release from human PBMCs treated with the HIV-1 isu peptide. While IL-10 secretion peaked after 15 h, IL-6 secretion continued to increase and had not yet reached its maximum at 24 h [10].

Additional insights were obtained using a cytokine array capable of detecting 105 cytokines and related molecules. Overall, 24 molecules were found to be upregulated (Table 2). The most strongly induced cytokines and chemokines included CCL3/CCL4 (MIP-1α/MIP-1β), CCL20 (MIP-3α), CXCL1 (GROα), IL-1β, and RANTES (CCL5). Notably, a similar cytokine profile has previously been reported following incubation of human donor PBMCs with the HIV-1 isu peptide [10] or the transmembrane envelope protein of HERV-K produced in yeast [11] (Table 2). IL-6 was readily detected by the cytokine array; however, IL-10 was not identified, despite the substantial increase in IL-10 secretion measured by ELISA (Figure 3). This discrepancy suggests a technical limitation of the array, potentially resulting from insufficient performance of the membrane-bound IL-10 capture antibody or the corresponding detection antibody used in the assay.

CCL3 and CCL4 (MIP-1α and MIP-1β) were significantly upregulated. These small (8–10 kDa) proinflammatory chemokines belong to the CC chemokine subfamily and are primarily produced by monocytes and macrophages [53]. They play key roles in leukocyte chemotaxis and migration across epithelial barriers [54]. Their biological effects are mediated through G protein-coupled chemokine receptors (CCRs), which are expressed on lymphocytes as well as monocytes/macrophages.

The release of CCL20 (MIP-3α) was likewise increased. CCL20 is unique among chemokines in that it is the only known ligand for CC chemokine receptor 6 (CCR6), a characteristic it shares with the antimicrobial β-defensins [55]. Defensins are small, cysteine-rich peptides with antimicrobial and antiviral properties that also participate in immune regulation [56,57]. Beyond their direct antimicrobial functions, defensins exert immunomodulatory effects by promoting chemotaxis and regulating cytokine production, including the induction of IL-1β, IL-6, IL-8, and TNF-α. In this context, it is noteworthy that a relationship between defensins and the HIV-1 isu peptide has previously been proposed [58]. The active region of the HIV-1 isu peptide was found to resemble both the pseudosubstrate regulatory domain of protein kinase C and a sequence present in cyclic immune peptides such as defensins. Moreover, defensins are capable of inserting into lipid bilayers, and the HIV-1 gp41-derived isu peptide has likewise been shown to associate with liposomes and lymphocyte membranes [58]. The CCL20–CCR6 ligand–receptor axis plays a critical role in the recruitment of immature dendritic cells, effector and memory T cells, and B cells, thereby contributing to the coordination of innate and adaptive immune responses.

Another molecule whose release was significantly increased was chemokine (C-X-C motif) ligand 1 (CXCL1), formerly known as growth-regulated oncogene-α (GROα), GRO1 oncogene, or melanoma growth-stimulating activity-α (MGSA-α). CXCL1 functions as a potent chemoattractant for several immune cell types, particularly neutrophils [59]. Similar to IL-8 (CXCL8), CXCL1 exerts its biological effects through binding to the chemokine receptor CXCR2, leading to activation of intracellular signaling pathways including phosphatidylinositol-3-kinase γ (PI3Kγ)/ serine/threonine kinase Akt (also known as protein kinase B or PKB), extracellular signal-regulated kinases 1 and 2 (ERK1/2), and phospholipase Cβ (PLCβ).

Two additional cytokines upregulated by p15E were IL-1β and CCL5 (RANTES, regulated upon activation, normally T-cell expressed and secreted). IL-1β is primarily produced by activated monocytes and macrophages and serves as a key mediator of inflammatory responses [60]. Elevated levels of IL-1β have been detected in the lymphoid tissues of HIV-infected individuals [60]. CCL5 is predominantly produced by T cells and monocytes [61]. Upon binding to its receptor CCR5, CCL5 activates phosphoinositide 3-kinase (PI3K), which subsequently phosphorylates and activates Akt. The concurrent suppression of MHC class II expression and reduction of cytotoxic cell activity, effectors of allograft rejection, indicates that the net immunological effect of p15E remains consistent with a reduction in adaptive immune reactivity [62,63].

Another molecule found to be upregulated by p15E was the urokinase-type plasminogen activator receptor (uPAR) (Table 2). uPAR is a cell-surface receptor for urokinase-type plasminogen activator (uPA), a serine protease involved in fibrinolysis, cell adhesion, migration, and tissue remodeling. Cleavage of uPAR by uPA generates soluble receptor fragments with distinct biological activities. These fragments can interact with members of the formyl peptide receptor (FPR) family; for example, the uPAR fragment comprising amino acids 88–95 binds to FPR1, whereas the fragment spanning residues 84–95 activates both FPR2 and FPR3 in basophils [64].

### 3.2. Influence of p15E on Gene Expression, Relationship with N-Formyl-Methionyl-Leucyl-Phenylalanine (fMLP)

The most comprehensive data were obtained by comparing gene expression profiles of PBMCs co-cultured with 293T cells expressing p15E to those of PBMCs co-cultured with wild-type 293T cells. This analysis revealed substantial transcriptional changes, with approximately 800 genes upregulated and 930 genes downregulated. These findings are consistent with the cytokine secretion and gene expression data described above, further supporting the immunomodulatory activity of p15E. Notably, similar transcriptional alterations were previously reported in studies investigating the effects of the HIV-1 immunosuppressive (isu) peptide [10] and the transmembrane envelope protein of HERV-K produced in yeast [11] on gene expression in human donor PBMCs (Table 3 and Table 4).

As previously observed for the HIV-1 isu peptide and the TM envelope protein of HERV-K, matrix metalloproteinase 1 (MMP-1) was among the most strongly upregulated genes (Table 3). MMP-1 is a zinc-dependent endopeptidase that degrades collagen and is produced by a variety of cell types, including activated macrophages and T lymphocytes. More broadly, MMPs play important roles in immune regulation and signaling by processing cytokines, chemokines, and extracellular matrix (ECM) components, thereby modulating the magnitude and duration of immune responses [65,66,67,68].

Increased MMP-1 expression is characteristic of classically activated (M1) macrophages, which are induced by Toll-like receptor ligands and proinflammatory cytokines such as TNF-α, IFN-γ, and IL-1. In vitro-differentiated M1 macrophages exhibit elevated expression of several MMPs, including MMP-1, MMP-3, MMP-7, MMP-10, MMP-14, and MMP-25, accompanied by reduced expression of tissue inhibitor of metalloproteinases-3 (TIMP-3) [68]. TIMP-3 belongs to a family of four TIMPs that regulate MMP activity, and the balance between MMPs and TIMPs is critical for maintaining ECM integrity and tissue homeostasis [69]. Consistent with this concept, elevated MMP-1 levels may contribute to the enhanced collagenolytic activity characteristic of M1 macrophages [66].

Beyond their roles in ECM remodeling, MMPs regulate inflammatory signaling through the proteolytic processing of cytokines and chemokines. Several MMPs, including MMP-1, MMP-2, MMP-3, MMP-9, MMP-12, MMP-14, MMP-15, and MMP-17, can cleave membrane-bound proTNF-α to generate biologically active TNF-α [67]. Likewise, MMPs can both activate pro-IL-1β and inactivate mature IL-1β through further proteolytic cleavage, thereby exerting both positive and negative regulatory effects on inflammation [70,71]. In addition, chemokines such as CCL2 (MCP-1), CCL8 (MCP-2), and CCL13 (MCP-4) can be processed by MMP-1, MMP-3, and MMP-8 to generate receptor antagonists with altered biological activities [72].

Interestingly, several MMPs, including MMP-1, MMP-2, MMP-3, MMP-10, MMP-13, and MMP-14, contain nuclear localization sequences that enable their translocation to the nucleus, suggesting functions beyond extracellular proteolysis [73]. MMP-1 expression can be induced by multiple cytokines, including IL-1, IL-4, IL-5, IL-6, IL-8, and IL-10 [74,75]. Since both IL-6 and IL-10 were upregulated in response to p15E, their increased production may contribute to the observed induction of MMP-1.

TREM1 (triggering receptor expressed on myeloid cells 1) was also found to be upregulated. TREM1 is a transmembrane protein belonging to the immunoglobulin (Ig) superfamily that is constitutively expressed on peripheral blood monocytes and neutrophils. Its expression is further enhanced by Toll-like receptor (TLR) ligands. Activation of TREM1 amplifies innate immune responses by promoting the production of proinflammatory cytokines and chemokines and by synergizing with TLR-mediated signaling pathways [76]. The upregulation of TREM1 reflects activation of innate immune signaling rather than enhancement of adaptive immunity, consistent with the broader pattern of retroviral immune dysregulation in which non-specific inflammatory activation and suppression of antigen-specific responses occur concurrently, as described for HIV-1 chronic infection.

Another upregulated gene was IL-24, a member of the IL-10 cytokine family. IL-24 is produced by a variety of immune cells, including activated lymphocytes, and has pleiotropic effects on immune regulation. In addition to its roles in inflammation and host defense, IL-24 has been reported to suppress the initial rounds of CD8^+^ T-cell expansion, thereby contributing to the regulation of adaptive immune responses [77].

Among the genes that were downregulated by p15E were TREM2, FCN1, CD36, FPR3, and CCL4. Notably, similar transcriptional changes were observed in our previous studies. In PBMCs exposed to the TM envelope protein of HERV-K, SEPP1, FCN1, FCN2, and TREM2 were among the most strongly downregulated genes, all of which are involved in innate immune functions [11]. Likewise, FCN1, SEPP1, TREM2, and CD36 were among the most strongly downregulated genes following treatment with the HIV-1 immunosuppressive (isu) peptide [10]. The recurrence of these alterations across different retroviral immunosuppressive proteins suggests the existence of a common mechanism targeting innate immune pathways.

TREM2 is a transmembrane receptor expressed predominantly on myeloid cells, including macrophages, monocytes, granulocytes, and dendritic cells [78]. Its extracellular domain recognizes a broad range of anionic ligands, including glycoproteins and lipids, whereas intracellular signaling is mediated through the adaptor proteins DNAX-activating protein 10 (DAP10) and DAP12 [79]. Although the functional consequences of TREM2 downregulation in the present study remain unclear, its repeated suppression by retroviral immunosuppressive proteins suggests that modulation of TREM2-dependent pathways may contribute to the alteration of innate immune responses. The downregulation of TREM2 may contribute to the pro-inflammatory component of the immune dysregulation observed, as TREM2 normally antagonizes TLR-mediated activation [79]. Paradoxically, TREM2 is also a marker of immunosuppressive tumor-associated macrophages (TAMs) [80], and its downregulation may reflect a shift away from regulatory macrophage polarization. Whether this represents a net pro- or anti-inflammatory effect in the context of xenotransplantation requires further investigation.

FCN1 (M-ficolin) is a pattern-recognition molecule of the complement system that is primarily expressed by myeloid cells. Structurally, it contains a collagen-like domain and a C-terminal fibrinogen-like domain and has been proposed to function as an elastin-binding plasma protein [81,82]. FCN1 is released from neutrophil granules following stimulation with N-formyl-methionyl-leucyl-phenylalanine (fMLP) or phorbol 12-myristate 13-acetate (PMA) [83]. Ficolins recognize acetylated carbohydrate structures on microbial pathogens, promote opsonization, and activate the lectin pathway of complement activation [84]. In our previous study on the HERV-K TM envelope protein, FCN2 (L-ficolin) was also markedly downregulated [11]. As emerging evidence suggests that L-ficolin deficiency is associated with increased susceptibility to respiratory infections [85], suppression of ficolin expression may impair early innate immune recognition and clearance of pathogens.

Taken together, the downregulation of FCN1, TREM2, and other genes involved in innate immune defense indicates that p15E interferes with key mechanisms responsible for the early detection and elimination of invading microorganisms. Such modulation of host immunity may facilitate viral persistence by weakening innate immune responses from the earliest stages of infection.

CD36 and FPR3 were also among the genes downregulated by p15E. CD36 is a multifunctional scavenger receptor that forms part of the non-opsonic CD36/αvβ3 receptor complex on macrophages and plays an important role in the recognition and phagocytic clearance of apoptotic cells and microbial pathogens [86]. Downregulation of CD36 may therefore impair macrophage-mediated phagocytosis and contribute to reduced innate immune surveillance.

FPR3 (formyl peptide receptor 3) is a seven-transmembrane G protein-coupled receptor involved in the regulation of chemotactic and inflammatory responses [87]. It is expressed by circulating monocytes, eosinophils, basophils, tissue macrophages, and dendritic cells, but not by neutrophils [88]. Although structurally related to FPR1, FPR3 differs functionally in that it does not respond to many classical FPR1 agonists, including the bacterial chemoattractant N-formyl-methionyl-leucyl-phenylalanine (fMLP, also known as fMLF) [89].

The downregulation of FPR3 is particularly noteworthy in light of earlier studies demonstrating interactions between retroviral immunosuppressive peptides and the formyl peptide receptor system. The hexapeptide LDLLFL, derived from the immunosuppressive (isu) domain of p15E (LQNRRGLDLLFLKE), was shown to be a potent inhibitor of fMLP-induced monocyte polarization, an early event in leukocyte chemotaxis [90]. Similar inhibitory effects were observed for the related retroviral peptides LDILFL from feline leukemia virus (FeLV) and LDLLFW from human T-cell leukemia viruses 1 and 2 (HTLV-1/-2). In addition to inhibiting monocyte polarization, LDLLFL suppressed fMLP-induced increases in intracellular calcium concentrations ([Ca^2+^]i), indicating interference with receptor-mediated signaling pathways [91]. Furthermore, the presence of LDLLFL shifted the fMLP dose–response curve toward higher ligand concentrations and reduced the binding of radiolabeled fMLP to neutrophils, suggesting direct competition with or modulation of formyl peptide receptor function [91].

Formyl peptides are generated during the degradation of bacterial and mitochondrial proteins and represent some of the earliest and most potent chemoattractant signals for activated leukocytes. Consequently, interference with formyl peptide receptor signaling may impair the recruitment and activation of monocytes and granulocytes at sites of infection [91]. In this context, it is noteworthy that a cleavage fragment of the urokinase plasminogen activator receptor (uPAR), comprising amino acids 84–95, has been reported to bind FPR3 and activate basophils [64]. Together with the observed upregulation of uPAR and downregulation of FPR3, these findings suggest that p15E may affect multiple components of the FPR signaling network, thereby modulating innate immune cell migration and activation.

In previous studies investigating the effects of the HIV-1 isu peptide [10] and the TM envelope protein of HERV-K [11], dehydrogenase/reductase 9 (DHRS9) was among the genes found to be significantly downregulated. In contrast, DHRS9 was detected in the present study. DHRS9 has been identified as a robust and specific biomarker of regulatory macrophages (Mregs), a distinct macrophage population with potent immunoregulatory properties that has been explored for use in cell-based immunosuppressive therapies for organ transplant recipients [92]. During the in vitro differentiation of human Mregs from CD14^+^ monocytes, DHRS9 expression increases progressively and is further enhanced by treatment with IFN-γ during the later stages of maturation [92]. In addition, DHRS9 expression in human tissues is highly responsive to inflammatory conditions, suggesting that it may serve as a sensitive marker of macrophage activation state and immune regulation [93].

### 3.3. Quantification of the Immunosuppressive Effect, Putative Receptors

The increased expression of p15E obtained with the new construct was associated with enhanced immunosuppressive activity, suggesting a higher abundance of biologically active molecules (Figure 2). Greater molecular density may also facilitate the formation of conformations required for efficient interaction with human PBMCs. Previous studies have shown that the immunosuppressive activity of retroviral ISU domains depends strongly on their structural presentation. Monomeric peptides are generally inactive, whereas BSA-conjugated peptides [10,12,13,14,15] or peptide homopolymers [10] exhibit immunosuppressive properties, both in vitro and in vivo [94]. Consistent with this, recombinant HIV-1 gp41 was approximately 700-fold more potent than homopolymers of the corresponding isu peptide in inducing IL-10 release [17]. Similar findings were reported for MuLV p15E, where p15E isolated from virus particles and present in complexes displayed substantially higher inhibitory activity (inhibition of proliferation of CTLL-2 cells) than recombinant protein or BSA-coupled synthetic peptides [13].

Although such direct comparisons had not been performed in vivo, immunosuppressive effects have been observed in vivo at very low doses. For example, 4 µg of the HIV-1 isu peptide significantly reduced PMA (phorbol 12-myristate 13-acetate)-induced macrophage recruitment into the murine peritoneal cavity [95]. This assay is a model for delayed-type hypersensitivity. In another study, as little as 0.2 ng of native p15E of MuLV inhibited macrophage accumulation during delayed inflammatory responses [96]. Together, these observations highlight the critical role of molecular conformation and multimerization in determining the biological activity of retroviral isu domains.

The cellular receptor(s) mediating the activity of the isu domain have not yet been identified, although several potential binding proteins have been reported [96,97,98,99,100]. The pronounced cross-species activity of retroviral TM envelope proteins and isu peptides suggests that the responsible receptor is evolutionarily conserved.

The fact that the isu peptide coated on porcine L23 cells is also able to induce IL-10 in human PBMCs may suggest that the peptide is binding to the receptor on the cells of this B cell line (Figure 9). Binding proteins for the ectodomain of gp41 of HIV-1 [97,98] and the isu peptide of HIV-1 [99,100] have been described on human B cells. Recombinant p15E of FeLV was also shown to bind to a human B cell line [101]. With increasing amounts of the peptide, the amount of released IL-10 increased, whereas higher amounts again reduced the IL-10 release, suggesting that an optimal amount of coated peptide is required for the highest release of IL-10. This result also indicates that the ISU domain is the functional region of the TM protein responsible for its immunomodulatory activity.

### 3.4. Implications for Xenotransplantation

The primary objective of the present study was to demonstrate that the expression of the TM envelope protein p15E of PERV on the surface of cells has an immunosuppressive effect. This strategy can be used to express p15E on a xenotransplant in order to prevent or at least reduce rejection. This could significantly reduce the need for systemic pharmacological immunosuppression. Such an approach has the potential not only to improve xenotransplant survival but also to enhance the overall safety of transplantation. A reduction in the use of immunosuppressive drugs would be expected to decrease systemic immune suppression and consequently lower the risk of reactivation of latent human viruses, including human cytomegalovirus (HCMV). Furthermore, it could reduce susceptibility to opportunistic infections and diminish the incidence of post-transplant malignancies. Cancer represents a major cause of morbidity and mortality in transplant recipients. In allotransplant recipients, malignancies account for approximately 10–30% of deaths, and tumors often exhibit a more aggressive clinical course than in the general population [102]. The high incidence of post-transplant de novo malignancies has been attributed to multiple factors, including impaired immune surveillance, reactivation of oncogenic viruses, chronic immune stimulation, the direct carcinogenic effects of immunosuppressive drugs, pre-existing cancer risk factors, and pathological conditions associated with end-stage organ disease [103].

Recently, other viral immunomodulatory proteins have also been expressed in porcine L23 cells, including the human adenovirus protein E3/49K and the HCMV protein pUL11 [104]. Both proteins interact with the human receptor-type protein tyrosine phosphatase CD45, a key regulator of signaling pathways downstream of the T-cell and B-cell antigen receptors [104,105,106,107]. Expression of E3/49K on L23 cells significantly reduced the cytotoxic activity of human T lymphocytes and NK cells, as well as B-cell activation and antibody production [103]. In addition, human PBMCs co-incubated with E3/49K-expressing L23 cells exhibited reduced expression of the proinflammatory cytokines TNF-α, IFN-γ, and IL-6, accompanied by increased expression of the anti-inflammatory cytokines IL-4 and IL-10. In contrast, expression of pUL11 did not produce comparable effects under the same experimental conditions [103]. These findings provide another example of how transgenic expression of viral immunomodulatory genes may be used to protect porcine xenotransplants. However, the use of the PERV TM envelope protein p15E may offer a distinct advantage. Because PERV sequences are integrated into the genome of all pigs, pigs are naturally tolerant to p15E and do not mount antibody responses against this protein [38]. In contrast, antibodies against species D adenoviruses are relatively common in humans [108], and because E3/49K is secreted and accessible in the extracellular environment, it could potentially become a target of antibody responses.

Therefore, the expression of the PERV transmembrane protein p15E represents a promising strategy to prevent xenotransplants from rejection or at least reduce the need for pharmacological immunosuppression, simultaneously reducing the adverse effects associated with long-term immunosuppressive therapy, including opportunistic infections, reactivation of latent human viruses, and the development of post-transplant malignancies.

## 4. Materials and Methods

### 4.1. Wild-Type 293T Cells, p15E-Expressing 293T Cells, PERV-Producing 293T Cells, and Human Peripheral Blood Mononuclear Cells (PBMCs)

Human embryonic kidney (HEK) 293T cells, designated as wild-type (wt) 293 cells, p15E-expressing 293T cells, and PERV-producing 293T cells were cultured in Dulbecco’s modified Eagle medium (DMEM) (Thermo Fisher Scientific, Waltham, MA, USA) supplemented with 10% heat-inactivated fetal bovine serum (Corning, Oneonta, NY, USA). Penicillin and streptomycin were not added to the culture medium.

p15E-expressing 293T cells were generated in this study; details are described in Section 4.3 and Section 4.4.

Human 293T cells were infected with a PERV-A/C recombinant originally isolated from pig PBMCs [41]. The virus had been serially passaged several times in human 293 cells, resulting in increased replication rates and an expanded number of repeat elements within the long terminal repeat (LTR) of the integrated provirus [31]. Recent analysis of the viral LTR showed that its length remained intermediate between those of PERV-3° and PERV-5° [39]. All cell cultures were routinely tested for mycoplasma contamination and were confirmed to be mycoplasma-free.

### 4.2. L23 Cells

The porcine peripheral blood B cell line L23 [45] was obtained from the European Collection of Cell Cultures. L23 is a B cell subline of the lymphoblastoid cell line B1 from a dd haplotype miniature swine, with a normal karyotype and producing immunoglobulin. No reverse transcriptase activity was found in the supernatant of L23 cells, indicating that they did not release retroviruses, whereas other sublines from the same lymphoblastoid cell line B1 did, and viral particles were shown by electron microscopy [45]. The L23 line was grown in Roswell Park Memorial Institute 1640 medium (RPMI 1640, PAN-Biotech, Aidenbach, Germany) supplemented with 10% heat-inactivated FBS, 2mM L-glutamine (PAN-Biotech, Aidenbach, Germany), 1mM sodium pyruvate (Cytiva, Marlborough, MA, USA), and 0.05 mmol/L β-mercaptoethanol (Sigma-Aldrich, St. Louis, MO, USA).

### 4.3. PCR-Based Assay to Detect Mycoplasma

Conventional PCR was used to detect mycoplasma. Cell culture supernatant was directly used as the template, with primers GGGAGCAAACAGGATTAGATACCCT and TGCACCATCTGTCACTCTGTTAACCTC, Dream Taq Green buffer (Thermo Fisher Scientific, Waltham, MA, USA), 10 mM dNTP Mix (Thermo Fisher Scientific, Waltham, MA, USA), and Dream Taq DNA polymerase (Thermo Fisher Scientific, Waltham, MA, USA). Thermal cycling was carried out as follows: 2 min initial denaturation at 95 °C, followed by 35 cycles of amplification (15 s at 95 °C, 20 s at 52 °C, 30 s at 72 °C), and 1 min final extension at 72 °C. PCR products were analyzed by 1% agarose gel electrophoresis.

### 4.4. Cloning of the Expression Construct

For stable surface expression of p15E of PERV, a γ-retroviral bicistronic expression vector was constructed based on the mbIL-2 membrane-anchoring design [40]. The cassette encodes, in tandem: the signal peptide encoded in the ENV gene of PERV; the p15E sequence comprising the N-terminal helix, C-terminal helix, and isu domain (with one of three cysteines substituted by serine to prevent intermolecular disulfide bonds while preserving the intramolecular fold); a triple FLAG epitope tag DYKDHDG-DYKDHDI-DYKDDDDK for detection; a hinge region; the transmembrane and anchor domains of MHC class I for correct surface tethering; an IRES; and ΔLNGFR (CD271) as a transduction marker, flanked by retroviral LTRs. 293T cells were transduced with γ-retroviral particles encoding this construct. Proviral integration was confirmed by flow cytometric detection of ΔLNGFR surface expression, which co-reports p15E transgene integration (Figure 1).

### 4.5. Generation of the p15E-Expressing 293T Cells

γ-retroviral particles were produced by transient transfection of 293T packaging cells with the transfer vector together with gag-pol and envelope plasmids. Viral supernatant was harvested at 48 and 72 h post-transfection. To generate p15E-expressing cells, 293T cells were transduced in the presence of protamine sulfate (4 µg/mL) with γ-retroviral particles encoding p15E co-expressed with the IRES-driven transduction marker ΔLNGFR (CD271). Cells were spinoculated at an MOI of 25 (800× *g*, 37 °C, 1 h). Forty-eight hours post-transduction, ΔLNGFR expression was quantified by flow cytometry, and ΔLNGFR^+^ (p15E^+^) cells were purified by FACS.

### 4.6. Flow Cytometry to Detect p15E Expression

Flow cytometry was used to analyze the expression of p15E protein on the cell surface of 293T cells. 293T cells, PERV p15E protein-expressing 293T cells, and PERV-producing 293T cells were harvested at around 90% confluence and washed twice with phosphate-buffered saline (PBS). Cells were detached with 0.05% trypsin for 30 s at room temperature and neutralized with complete culture medium. Each fluorescence-activated cell sorting (FACS) sample contained 6 × 10^5^ cells; duplicates (1.2 × 10^6^ cells) were analyzed. Cells were centrifuged at 200× *g* for 2 min (centrifuge 5425 R, Eppendorf, Hamburg, Germany) and resuspended in 10 mL DMEM with 10% heat-inactivated FBS (Corning), centrifuged again at 200× *g* for 2 min, incubated with fluorescence-activated cell sorting (FACS) blocking buffer consisting of PBS supplemented with 2 mM ethylenediaminetetraacetic acid (EDTA), 0.02% sodium azide (NaN_3_) and 10% heat-inactivated horse serum for 20 min. Cells were incubated with goat anti-p15E serum #355 [43] (diluted 1:100) in FACS buffer (PBS supplemented with 2 mM EDTA, 0.02% NaN_3_ and 1% heat-inactivated horse serum) for 30 min. Cells were washed twice with FACS buffer. Cells were then incubated with secondary antibody, donkey anti-goat IgG (H+L)-FITC (Invitrogen, Ballenger Creek, MD, USA), at a dilution of 1:500 for 30 min. Cells were washed once, resuspended in 520 µL FACS buffer, and distributed into two wells of a 96-well U-bottom plate. Analysis was performed on a CytoFLEX flow cytometer (Beckman Coulter, Brea, CA, USA), and data were analyzed using CytExpert Version 2.1 (Beckman Coulter, Brea, CA, USA).

As the construct of PERV p15E protein also contains a FLAG DYKDHDG-DYKDHDI-DYKDDDDK tag and an ΔLNGFR reporter, additional flow cytometry was performed with the same batch. Cells were stained with a mixture of phycoerythrin (PE)-labeled rat anti-FLAG antibody (BioLegend, San Diego, CA, USA, RRID: AB_2563147) and allophycocyanin (APC)-labeled mouse anti-human CD271 antibody (NGFR) (BioLegend, San Diego, CA, USA, RRID: AB_10639737), each diluted 1:200 in FACS buffer. In one of the laboratories, FLAG expression was detected by indirect immunofluorescence using the mouse mAb M2 antibody (Sigma-Aldrich, St. Louis, MO, USA, RRID: AB_259529) as primary antibody followed by FITC-conjugated goat anti-mouse IgG plus IgM (Jackson ImmunoResearch, Cambridgeshire, UK, RRID: AB_2338612).

### 4.7. Isolation of Peripheral Blood Mononuclear Cells (PBMCs)

At the Institute of Virology in Berlin, PBMCs were isolated from buffy coats from human blood from anonymous donors and from a healthy co-worker at the institute. For purification, Ficoll–Hypaque density centrifugation using 50 mL Leucosep Tubes (Greiner Bio-One, Kremsmünster, Austria) was used according to the instructions of the manufacturer. The purified PBMCs were counted using a Neubauer Chamber (Marienfeld, Lauda-Königshofen, Germany), and 1.5 × 10^8^ PBMCs were frozen in cryopreserved tubes and stored at 150 °C in a freezing medium containing 90% FBS and 10% dimethyl sulfoxide (DMSO).

At the Transplant Laboratory in Hannover, PBMCs were isolated from discarded material of normal routine apheresis samples from anonymized donors.

PBMCs were cultured in RPMI 1640 PAN-Biotech, Aidenbach, Germany) supplemented with 10% heat-inactivated FBS, 2 mM L-glutamine (PAN-Biotech, Aidenbach, Germany) and 1 mM sodium pyruvate (Cytiva, Marlborough, MA, USA).

### 4.8. Ethics Declarations

At the Institute of Virology in Berlin, buffy coats from human blood from an anonymous donor were provided by Deutsches Rotes Kreuz, Blutspendedienst Nord-Ost, Berlin. In the case where blood was taken from an institute co-worker, a consent form for blood collection for scientific purposes was signed by the volunteer. The use of human blood has been approved by the ethics committee at the Medical Faculty of Humboldt University Berlin. At the Transplant Laboratory in Hannover, PBMCs were isolated from discarded material of normal routine apheresis samples obtained from the Department of Transfusion Medicine of the Hannover Medical School. Samples were anonymized and could not be assigned to an individual donor. The local ethics committee of Hannover Medical School approved this procedure. All methods were carried out in accordance with DFG guidelines of Good Scientific Practice.

### 4.9. Co-Cultivation of PBMCs with wt 293T Cells and p15E-Expressing 293T Cells

293T cells, PERV p15E protein-expressing 293T cells, and PERV-producing 293T cells, all cultured in DMEM and heat-inactivated FBS, were used at 50% to 60% confluence. Cells were washed twice with PBS, detached with 0.05% trypsin for 30 s at room temperature, and neutralized with complete culture medium, counted using a Neubauer chamber, and seeded into a 96-well plate, with 1.5 × 10^4^ cells per well in 200 µL of DMEM with 10% heat-inactivated FBS. Cells were incubated overnight at 37 °C in a humidified chamber with 5% CO_2_. The following day, 100 µL of the medium was removed and replaced with 100 µL of PBMCs (3 × 10^5^ cells per well) in RPMI 1640 with 10% heat-inactivated FBS, 2 mM L-glutamine, and 1 mM sodium pyruvate. Cells were co-incubated overnight at 37 °C in a humidified chamber with 5% CO_2_. PBMCs had been thawed from −150 °C and cultured overnight prior to the co-culture.

### 4.10. IL-6 and IL-10 ELISAs

To quantify cytokine IL-10 in the co-culture supernatants, the Human IL-10 ELISA Kit II or the Human IL-10 ELISA Set; to quantify cytokine IL-6, the Human IL-6 ELISA Kit II (all BD Biosciences, San Jose, CA, USA) were used. 100 µL of supernatant was collected after 24 h of incubation, after centrifuging at 2000× *g* for 10 min (centrifuge 5425 R, Eppendorf, Hamburg, Germany). Following the instructions of the supplier, 50 µL of ELISA diluent was added to each well. In parallel, 100 µL of each standard and diluted sample (samples were diluted in Standard/Sample Diluent prior to ELISA (IL-6: 1:100; IL10: 1:8)) were pipetted into appropriate wells and incubated for 2 h. ELISA was performed in duplicate. After washing five times, 100 µL of prepared working detector was added to each well and incubated for 1 h. After washing seven times, 100 µL of 3,3′,5,5′-tetramethylbenzidine (TMB) one-step substrate reagent was added to each well and incubated for 30 min in the dark. 50 µL of stop solution was added. Absorbance was measured using a TriStar LB 941 microplate reader (Berthold Technologies, Bad Wildbad, Germany) set to 450 nm. According to the protocol of the Human IL-10 ELISA Set, coating buffer (0.1M sodium carbonate, pH 9.5), assay diluent (PBS with 10% FBS, pH 7.0), and wash buffer (PBS with 0.05% Tween-20) were prepared before the experiment. The ELISA plate (Sarstedt, Nümbrecht, Germany) was incubated with a mixture of capture antibody and coating buffer at a 1:250 ratio overnight at 4 °C. After three washes, the plate was blocked with 200 µL assay diluent for 1 h at RT. Following another three washes, 100 µL of standards and samples were added to the plate and incubated for 2 h at RT. After five washes, 100 µL of working detector (detection antibody and Sav-HRP) was added to each well and incubated for 1 h at RT. Following seven washes, 100 µL TMB substrate was added and incubated for 30 min at RT. The reaction was stopped by adding 50 µL of stop solution. Absorbance was measured at 450 and 570 nm using Biotek Synergy H1 Hybrid Reader (BioTek Instruments GmbH, Bad Friedrichshall, Germany). The ΔOD (OD450 − OD570) values were calculated, and cytokine concentrations were determined from the standard curve by BioTek Gen5 software (version 5.3).

### 4.11. Intracellular Cytokine Detection in PBMC by Flow Cytometry

A new cytokine assay was developed, based on intracellular cytokine staining and flow cytometry. PBMCs from healthy human donors were co-cultivated with wt 293T cells, PERV p15E-expressing 293T cells, and PERV-producing 293T cells at 37 °C. Three protocols were performed: (i) 24 h plus five hours in the presence of Brefeldin A, (ii) overnight incubation followed by five hours with Brefeldin A (total incubation time of 24 h), and (iii) a six-hour incubation including the final five hours with Brefeldin A. Lipopolysaccharide from *Escherichia coli* O26:B6 (LPS, Merck KGaA, Darmstadt, Germany; 1 µg/mL) was used as a positive control; it was added only for one h. Brefeldin A (eBioscience Inc., San Diego, CA, USA; 3.0 µg/mL) was always added during the last five hours. After incubation, the cells were washed in FACS buffer (PBS supplemented with 1% heat-inactivated FBS and 2mM EDTA) and stained for 20 min on ice with APC-labeled anti-CD14 antibody (clone TÜK4, Miltenyi Biotec, Bergisch Gladbach, Germany; RRID:AB_244301, 1:50), PerCP-labeled anti-CD4 (clone RPA-T4, BioLegend, San Diego, CA, USA; RRID:AB_893327, 1:20), Brilliant Violet-labeled anti-CD21 (clone B-ly4, BD biosciences San Jose, CA, USA; RRID:AB_2739918, 1:100) and fixable viability dye eFluor 780 (eBioscience; 1:1000). Cells were washed with FACS buffer once, then resuspended in 100 µL intracellular fixation buffer (eBioscience) and incubated at 4 °C overnight. Cells were washed twice with 200 µL permeabilization buffer (eBioscience Inc., San Diego, CA, USA) and stained with FITC-labeled anti-IL-6 (clone MQ2-13A5, BioLegend, RRID:AB_315151; 1:100), Pacific Blue-labeled anti-TNF-α (clone Mab11, BioLegend, RRID:AB_528965; 1:100) and PE-labeled anti-IL-10-PE (clone JES3-9D7, eBioscience, RRID:AB_466179; 1:100) for 1 h at room temperature. PE-labeled rat IgG1 κ (clone 3.6.2.8.1., Life Technologies Corp., Carlsbad, CA, USA, RRID:AB_470117; 1:100) was used as an isotype control. The samples were washed twice with permeabilization buffer and acquired on a FACSCanto II flow cytometer (BD Biosciences). Data were analyzed with FlowJo software version 10.10.1 (BD Biosciences).

### 4.12. Membrane-Based Cytokine Array

Using the Proteome Profiler Human XL Cytokine Array Kit (BD Biosciences, San Jose, CA, USA), 105 different cytokines were analyzed. Supernatants were collected after 24 h of co-culture and centrifuged at 2000× *g* for 10 min to remove the cell pellets. 2 mL of array buffer 6 blocking buffer was pipetted into each well of the 4-well multi-dish. Each membrane was placed in a separate well and incubated for 1 h on the rocking platform shaker. Array buffer 6 was aspirated, and a mixture of 200 µL of samples and 1.3 mL of array buffer 6 was added and incubated overnight at 4 °C on a rocking platform shaker. Membranes were removed and placed into individual plastic containers with 20 mL of wash buffer. Membranes were washed three times with wash buffer. 30 μL of detection antibody cocktail to 1.5 mL of array buffer 4 and 6 was added to each array and incubated for 1 h on a rocking platform shaker. Each array was washed three times. 2 mL of streptavidin-labeled horseradish peroxidase (HRP) was added to each well and incubated for 30 min. Membranes were washed three times; 1 mL of the freshly prepared chemiluminescent reagent mixture was evenly applied to each membrane and incubated for 1 min. Signals were analyzed using the Chemi-Smart 5100 imaging system (peqlab Biotechnologie GmbH, Erlangen, Germany).

### 4.13. RNA Extraction

In order to analyze the expression of cytokines, RNA was isolated using the RNeasy Kit (Qiagen, Hilden, Germany) and DNase (New England Biolabs, Frankfurt am Main, Germany) treatment to remove cellular DNA.

### 4.14. Detection of Cytokine Expression by Real-Time RT-PCR

To analyze expression of IL-6, IL-10, and TNF-α, a real-time RT-PCR for each of the factors was established, using primers and probes (Table 5). RNA was isolated from pooled culture wells, treated with DNase I and analyzed using human GAPDH as a control: No-ROX one-step kit (Meridian Life Science, Memphis, TN, USA), 30 min reverse transcription at 50 °C, 5 min denaturation at 95 °C, 45 cycles (10 s at 95 °C, 20 s 54 °C, 15 s 72 °C). A qTOWER3 qPCR-Thermocycler (Analytik Jena, Jena, Germany) was used in all experiments. Gene expression was calculated according to the 2^−ΔΔCt^ method [109]. Three Ct values were measured for each cytokine and the housekeeping gene GAPDH. The columns show the fold change 2^−ΔΔCt^ value. From the Ct values measured for the cytokine and the control GAPDH, ΔCt = Ct_cytokine_ − Ct_GAPDH_ was calculated. From this, the mean ΔCt and the SD ΔCt were calculated, and ΔΔCt = ΔCt_experiment_ − ΔCt_control_; the Fold Change = 2^−ΔΔCt^, the error bar lower = 2^−(SD ΔC),^ and the error bar upper = 2^(SD ΔCt)^.

### 4.15. CD107a Assay

CD56^+^CD3^−^ NK cells were used as the cytotoxic effector population. The cells were obtained by MACS-mediated depletion of CD4^+^CD8^+^HLA-DR^+^CD14^+^ cells from PBMCs. Usually, the great majority (67 to 80%) of the enriched cell population expressed a typical CD56^+^CD3^−^ NK cell phenotype. In some experiments, cells expressing a CD56^+^CD3^+^ phenotype (up to 12%) were present. NK cells (2 × 10^5^ cells) were co-cultured for two hours with 2 × 10^5^ wt 293T cells or 2 × 10^5^ 293T cells expressing p15E. Cytotoxic activity against 293T target cells was monitored by assessing CD107a expression (degranulation) on gated CD56^+^ CD45^+^ NK cells.

### 4.16. Analysis of the Gene Expression (Microarray)

To compare the expression of genes in human PBMCs co-incubated with 293T cells expressing p15E with the gene expression in PBMCs co-cultivated with wt 293T cells, three samples of each combination were co-cultivated for 24 h. Afterwards, the cells were washed with 1 mL of RNase-free PBS buffer and TRIzol buffer (Invitrogen, Ballenger Creek, MD, USA). The cell suspension was aspirated using a pipette, ensuring disruption of any visible clumps until the suspension achieved clarity. The suspension was stored at −80 °C and shipped on dry ice. RNA isolation from each of the six samples and their RNA sequencing was performed by Biomarker Technologies (BMK) GmbH, Münster, Germany. Briefly, purity, concentration, and integrity of the RNA sample were examined by NanoDrop, Qubit 2.0, and Agilent 2100. mRNA was isolated by Oligo(dT)-attached magnetic beads. mRNA was then randomly fragmented in fragmentation buffer, and first-strand cDNA was synthesized with fragmented mRNA as a template and random hexamers as primers, followed by second-strand synthesis with the addition of PCR buffer, dNTPs, RNase H, and DNA polymerase I. Purification of cDNA was performed with AMPure XP beads. Double-stranded cDNA was subjected to end repair. Adenosine was added to the ends and ligated to adapters. AMPure XP beads were applied to select fragments within the size range of 300–400 bp. cDNA library was obtained by certain rounds of PCR on cDNA fragments. The qualified library was sequenced on a high-throughput sequencing platform in PE150 mode.

Reference genome Homo_sapiens. GRCh38_release104.genome.fa was predefined for the analysis. HISAT2 was used for mapping RNA-seq reads, which is a more advanced version of TopHat2/Bowtie2. HISAT2 uses a Burrows-Wheeler Transform and Ferragina-Manzini (FM) index-based search. HISAT2 uses one global graph FM index (GFM) to represent the general population, as well as small indexes (local indexes) combined with several alignment strategies in order to achieve more efficient alignment. StringTie was applied to assemble the mapped reads. The algorithm is established based on optimality theory. It utilizes a novel network flow algorithm as well as an optional de novo assembly step to assemble and quantify transcripts representing multiple spliced variants for each gene locus.

### 4.17. Synthetic Isu Peptide of PERV

A synthetic peptide corresponding to the isu sequence of PERV (H-LQNRRGLDLLFLKEGGL-OH) was produced by Intavis Peptide Services, Tübingen, Germany. Its mass according MALDI-MS was 1941.12 Da, and its purity was 97.4%. The peptide was dissolved in sterile, endotoxin-free PBS (Sigma-Aldrich, Schnelldorf, Germany).

### 4.18. Loading of L23 Cells with the Isu Peptide of PERV and Co-Culture with Human PBMCs

L23 cells (3 × 10^5^ cells or 1.5 × 10^5^ cells) seeded in 96-well plates were loaded with dilutions of the isu peptide, and after incubation for different time points 30 and 60 min), human PBMCs (3 × 10^5^ cells) were added to the treated or untreated L23 cells. The PBMCs were thawed the same day or pre-cultured overnight in RPMI 1640 culture medium with 10% FBS, and then added. After a co-incubation for 24 h, IL-10 and IL-6 were determined in the supernatant.

### 4.19. Statistical Analysis

Statistical analyses and graphical presentations were performed using GraphPad Prism (version 10.1.2), and raw data were processed using Microsoft Excel (Version 2608). qPCR data were calculated according to the 2^−ΔΔCt^ method [109]. Biological replicates were considered independent experimental units for statistical analysis, whereas technical replicates were averaged before statistical analysis. Statistical significance was assessed using a two-tailed paired *t*-test, with a *p*-value < 0.05 considered statistically significant. For GO and KEGG enrichment analyses, over-representation of differentially expressed genes (DEGs) was assessed using Fisher’s exact test. Enrichment factors were used to evaluate the degree of enrichment, and Q-values were calculated to account for multiple testing. Enriched terms and pathways were ranked according to their Q-values.

## 5. Conclusions

The TM protein of PERV expressed on the surface of 293T cells was shown to induce the production of IL-10 and IL-6, modulate the expression of additional cytokines, and alter the expression of hundreds of genes in co-cultivated human PBMCs, indicating an immunosuppressive effect on human immune cells. Furthermore, p15E expressed on 293T cells inhibited the activity of human NK cells. In addition, the isu peptide of PERV coated on porcine L23 cells, a B-cell line, induced increased expression of IL-10 in human PBMCs.

Overall, the immunosuppressive properties of PERV p15E and its isu peptide were comparable to those previously reported for the TM envelope proteins and corresponding isu peptides of several other retroviruses, including HIV-1. These findings further support the concept of exploiting p15E expression to reduce immune-mediated rejection of xenotransplants or, at the very least, to decrease the need for pharmacological immunosuppression. Such an approach could also lessen the adverse effects associated with long-term immunosuppressive therapy, including opportunistic infections, reactivation of latent viruses, and the development of post-transplant malignancies.

## Figures and Tables

**Table 1:** Results of the FACS-based cytokine assay measuring intracellular cytokines using specific antibodies, percentage of positive cells. n.d., not done.

	IL-10		IL-6		TNF-α	
Incubation Time (Hours)	24	6	24	6	24	6
PBMCs alone	n.d.	1.6	n.d.	13.8	n.d.	6.8
LPS	n.d.	19.9	n.d.	52.0	n.d.	89.8
293T cells	0.2	0.8	26.6	7.5	0.4	2.6
293T cells expressing p15E	30.8	15.8	85.8	18.7	92.2	76.6
PERV 293T	40.2	20.1	87.9	18.7	93.0	82.4

**Table 2:** Increase of cytokine release by human PBMCs co-incubated with 293T cells expressing p15E of PERV after 8 and 24 h or producing PERV after 24 h compared with the cytokine expression of human PBMCs incubated with 293T cells alone for 24 h. For comparison, published data obtained after incubation of HIV isu peptide [10] and HERV-K TM envelope protein [11] with human PBMCs for 24 h, but using a different cytokine array, were added.

Cytokine	Experiment 1 ^a^	Experiment 2 ^b^			HIV ^c^	HERV-K ^d^
	293T Cells p15E 24 h	293T Cells p15E 24 h	293T Cells p15E 8 h	293T Cells PERV 24 h		
Angiogenin (RNase 5)		+ ^e^	-	-		
CD147 (EMMPRIN)	++	+	+	+		
CXCL5 (ENA78)		++	+	++		
FGF-19		+	-	-		
G-CSF		+	-	-		yes
GM-CSF		+	+	+	yes	
CXCL1 (Groα)	++	+++	+	++	yes	yes
IL-1β		++	+	++	yes	
IL-1Ra	++	+	-	+		
IL-6		++	-	+	yes	yes
IL-8		+	+	+	yes	yes
IL-17		+	-	-		
MIF		+	+	+		
CXCL10 (IP10)		+	-	-		
CCL2 (MCP1)		+	+	+	yes	yes
CCL3/CCL4 (MIP-1α/MIP1β)	+++++	+++	++	++	yes	yes
CCL20 (MIP-3α)	+++++	++++	+	++++		
Osteopontin	+++	+	-	-		
CCL5 (RANTES)	++	+++	++	+++	yes	yes
PAI-1 (SERPIN E1)		+	+	+		
Thrombospondin-1	+	++	++	++		
TNF-α	++	++	++	+	yes	
uPAR	++	+	-	+	yes	yes
PECAM-1 (CD31)		+	+	-		

^a^, ^b^, two independent experiments; ^c^, results from Denner et al., 2013 [10]; ^d^, results from Morozov et al., 2013 [11]. ^e^, the number of + indicate the differences in the strength of the dot in the cytokine array compared with that from wt 293 cells.

**Table 3:** Immune system associated genes with the highest up-regulation in human donor PBMCs co-incubated with 293T cells expressing p15E in comparison to PBMCs co-incubated with wild-type 293T cells.

Gene	P ^a^	log2FC	Protein	Alternative Name	Position HIV-1 Peptide ^b^	Position HERV-K Protein ^c^
MMP-1	1	13.84	matrix metalloproteinase-1	interstitial collagenase	2	1
CSF3	5	10.85	colony stimulating factor 3	granulocyte colony-stimulating factor (G-CSF)	1	
IL6	10	9.13	interleukin 6 (IL-6)			2
CCL20	15	8.83	chemokine (C-C motif) ligand 20	macrophage inflammatory protein-3 (MIP3A)		39
PTGS2	17	8.83	prostaglandin-endoperoxide synthase 2	cyclooxygenase-2 (COX-2)	5	11
CXCL5	19	8.40	chemokine (C-X-C motif) ligand 5	ENA78	4	
CXCL1	21	8.20	chemokine (C-X-C motif) ligand 1	GROα. melanoma growth stimulating activity. alpha (MGSA-α)	7	7
IL1B	23	7.99	interleukin-1 beta (IL-1β)	lymphocyte activating factor	11	12
TNFAIP6	26	7.76	tumor necrosis factor-inducible gene 6 protein	TNF-stimulated gene 6 protein (TSG-6)		
CCL3L1	28	7.56	chemokine (C-C motif) ligand 3-like 1		40	
IL1A	31	7.20	interleukin-1 alpha (IL-1 α)	lymphocyte-activating factor (LAF)		3
TNFRSF11B	39	6.76	tumor necrosis factor receptor superfamily member 11B	osteoprotegerin (OPG)		
CXCL3	44	6.51	chemokine (C-X-C motif) ligand 3	macrophage inflammatory protein-2-beta (MIP2β)	18	20
CCL3	54	6.05	chemokine (C-C motif) ligand 3	macrophage inflammatory protein 1-alpha (MIP-1-α)		
TREM1	58	5.84	triggering receptor expressed on myeloid cells 1		9	6
CXCL8	59	5.80	interleukin 8 (IL-8)	chemokine (C-X-C motif) ligand 8. (CXCL8)	20	17
CXCL2	60	5.78	chemokine (C-X-C motif) ligand 2	macrophage inflammatory protein 2-alpha (MIP2-α). Growth-regulated protein beta (Gro-β)		
IL24	84	4.86	interleukin 24 (IL-24)	melanoma differentiation-associated 7 (mda-7)	6	13
CSF2	87	4.83	granulocyte-macrophage colony-stimulating factor (GM-CSF)	colony-stimulating factor 2 (CSF2)		
IL10	90	4.75	interleukin 10 (IL-10)			
TNFRSF9	101	4.28	tumor necrosis factor receptor superfamily member 9	CD137		
CCL4	110	3.99	chemokine (C-C motif) ligands 4	macrophage inflammatory proteins (MIP-1β)		
IL2RA	147	3.20	interleukin-2 receptor alpha chain	CD25		
CXCR5	187	2.67	C-X-C chemokine receptor type 5			
CXCL6	191	2.64	chemokine (C-X-C motif) ligand 6		21	41

^a^, position (P) in the full list of genes up-regulated in PBMCs co-incubated with 293T cells expressing p15E in comparison with PBMCs co-incubated with wild-type 293T cells; ^b^, position in the list of the fifty genes with the highest increase in expression upon incubation of human PBMCs with the HIV isu homopolymer [10], ^c^, position in the list of the fifty up-regulated genes with the highest increase in expression upon incubation of human PBMCs with the recombinant TM envelope protein of HERV-K [11].

**Table 4:** Immune system associated genes with the highest down-regulation in human donor PBMCs co-incubated with 293T cells expressing p15E in comparison to PBMCs co-incubated with wild-type 293T cells.

Gene	P ^a^	log2FC	Protein	Alternative Name	Position HIV-1 Peptide ^b^	Position HERV-K Protein ^c^
TREM2	10	−7.96	triggering receptor expressed on myeloid cells 2		5	6
FCN1	12	−7.41	ficolin-1	M-ficolin	1	2
CD36	24	−5.50	cluster of differentiation 36	granulocyte colony-stimulating factor (G-CSF)	1	18
FPR3	25	−5.47	N-formyl peptide receptor 3			
CCL24	31	−4.85	chemokine (C-C motif) ligand 24	myeloid progenitor inhibitory factor 2 (MPIF-2)		
HLA-DRA	343	−1.71	HLA class II histocompatibility antigen, DR alpha chain			
HLA-DRB5	416	−1.57	HLA class II histocompatibility antigen, DRB5 beta chain			
HLA-DRB1	474	−1.46	HLA class II histocompatibility antigen, DRB1 beta chain			
HLA-DPB1	616	−1.32	HLA class II histocompatibility antigen, DP(W2) beta chain			
HLA-DMB	677	−1.25	HLA class II histocompatibility antigen, DM beta chain			38

^a^, position (P) in the full list of genes up-regulated in PBMCs co-incubated with 293T cells expressing p15E in comparison with PBMCs co-incubated with wild-type 293T cells; ^b^, position in the list of the fifty genes with the highest increase in expression upon incubation of human PBMCs with the HIV isu homopolymer [10], ^c^, position in the list of the fifty up-regulated genes with the highest increase in expression upon incubation of human PBMCs with the recombinant TM envelope protein of HERV-K [11].

**Table 5:** Primers used for the analysis of cytokine expression.

Primer	Sequence	Reference	Accession Number	Localization
GAPDH fw	5′-GGCGATGCTGGCGCTGAGTAC-3′	Denner et al. [10]	NM 002046.7	338–358
GAPDH rev	5′-TGGTTCACACCCATGACGA-3′			468–486
GAPDH probe	5′-HEX-CTTCACCACCATGGAGAAGGCTGGG-BHQ-1-3′			379–403
IFN-γ fw	5′-GCATCGTTTTGGGTTCTCTTG-3′	Denner et al. [39]	NM 000619.3	163–183
IFN-γ rev	5′-AGTTCCATTATCCGCTACATCTG-3′			253–275
IFN-γ probe	5′-6-FAM-TCTGCTTCTTTTACATATGGGTCCTGGC-BHQ-1-3′			193–220
IL-6 fw	5′-GGTACATCCTCGACGGCATCT-3′	Denner et al. [10]	NM 000600.5	236–256
IL-6 rev	5′-GTGCCTCTTTGCTGCTTTCAC-3′			296–316
IL-6 probe	5′-6-Fam-TGTTACTCTTGTTACATGTCTCCTTTCTCAGGGCT-BHQ-1-3′			258–292
IL-10 fw	5′-CCACGCTTTCTAGCTGTT-3′	Denner et al. [10]	NM 000572.3	966–983
IL-10 rev	5′-CTCCCTGGTTTCTCTTCCTAA-3′			1058–1078
I-10 probe	5′-6-FAM-TCTTGTCTCTGGGCTT-BHQ-1-3′			1015–1030
MMP1 fw	5′-CATCCAAGCCATATATGGACG-3′	Denner et al. [10]	NM 002421.4	833–853
MMP1 rev	5′-tctggagagtcaaaattctct-3′			1423–1443
MMP1 probe	5′-6-FAM-CTGGGCTGTTCAGGGACAGAA-BHQ-1-3′			1112–1132
TNF-α fw	5′-GAGAAGCAACTACAGACCCC-3′	Denner et al. [39]	NM 000594.4	48–67
TNF-α rev	5′-CATGCTTTCAGTGCTCATGG-3′			176–195
TNF-α probe	5′-6-FAM-ACAACCCTCAGACGCCACATCC-BHQ-1-3′			76–97

## Data Availability

All data are included in this manuscript and the Supplementary Materials.

## Supplementary Materials

The following supporting information can be downloaded at: https://www.mdpi.com/article/10.3390/ijms27188219/s1.
