# Supplementary material for: The Transmembrane Envelope Protein of Porcine Endogenous Retroviruses Modulates Cytokine Release and Gene Expression in Human Immune Cells
Source: Int J Mol Sci. 2026 Sep 15;27(18):8219. doi: 10.3390/ijms27188219 (PMC13607933; doi:10.3390/ijms27188219)
Supplement: Supplementary file 1 [file ijms-27-08219-s001.zip › ijms-4517290-supplementary.pdf]

Table S1. Results of a FACS based cytokine assay (positively stained cells in %), using 29 h co-cultivation including 5 h incubation with Brefeldin A

PBMCs co-incubated with	IL-10	IL-6	TNFα	$CD14^-$	$CD4^+$ T cells	$CD19^+$ B cells	Mono-cytes	Source
PBMCs alone	1	0.9	0.8	56	39	15	16	Figure S1, A
LPS	10	49	70	55	37	16	14	Figure S1, B
LPS isotype antibody	0.3	0	0.2	53	38	15	15	Figure S1, C
293T cells	0.2	0.2	0	52	37	17	18	Figure S1, D
293T cells expressing p15E-	9	12	0.4	56	32	21	11	Figure S1, E
PERV 293T	4	7	0.6	57	32	17	18	Figure S1, F

Figure S1

A, PBMC

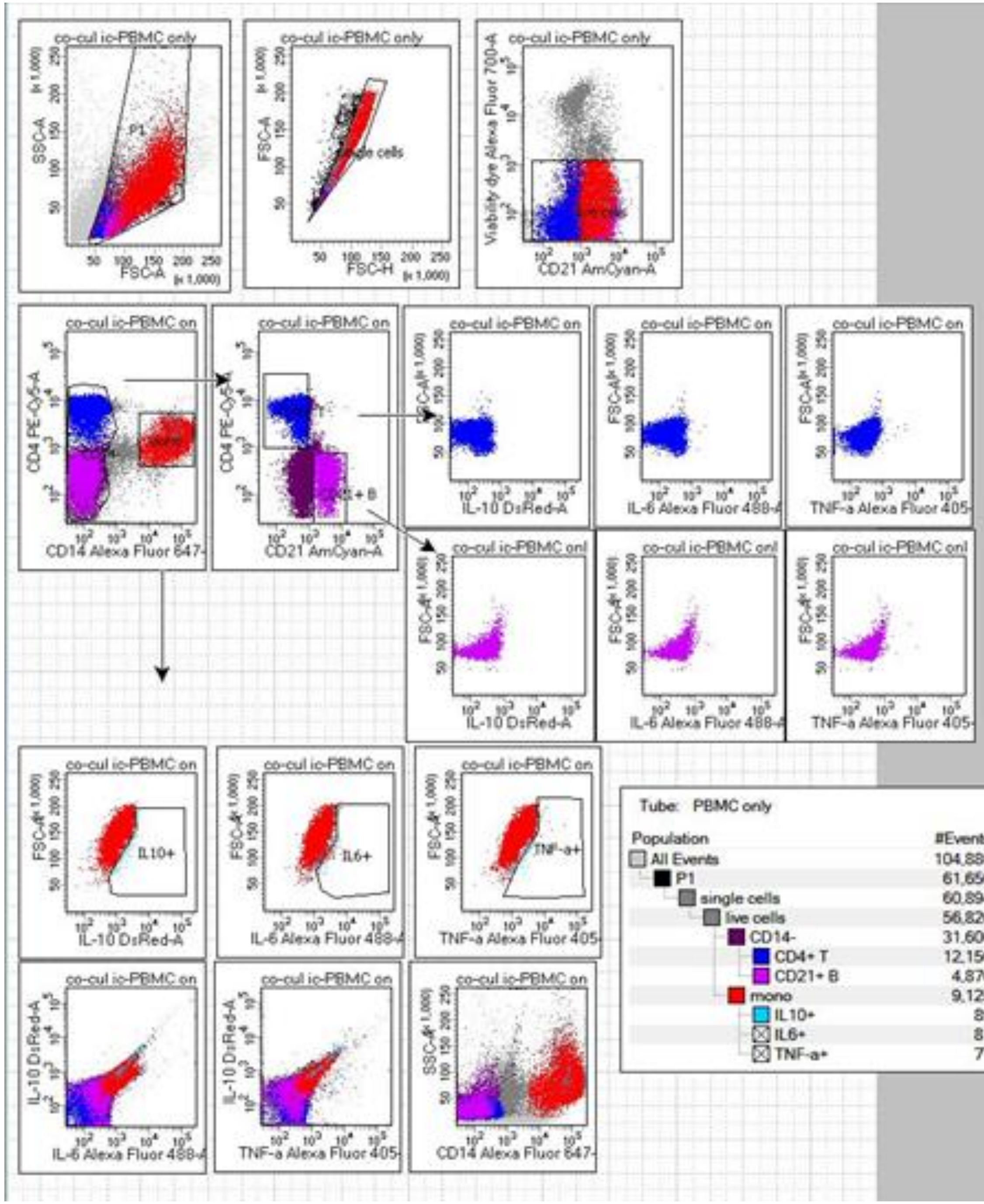

Tube: PBMC only

Population	#Events	%Parent	%Total
All Events	104,880	####	100.0
P1	61,650	58.8	58.8
single cells	60,894	98.8	58.1
live cells	56,820	93.3	54.2
CD14-	31,606	55.6	30.1
CD4+ T	12,156	38.5	11.6
CD21+ B	4,876	15.4	4.6
mono	9,125	16.1	8.7
IL10+	89	1.0	0.1
IL6+	81	0.9	0.1
TNF-a+	77	0.8	0.1

Figure S1

B, LPS

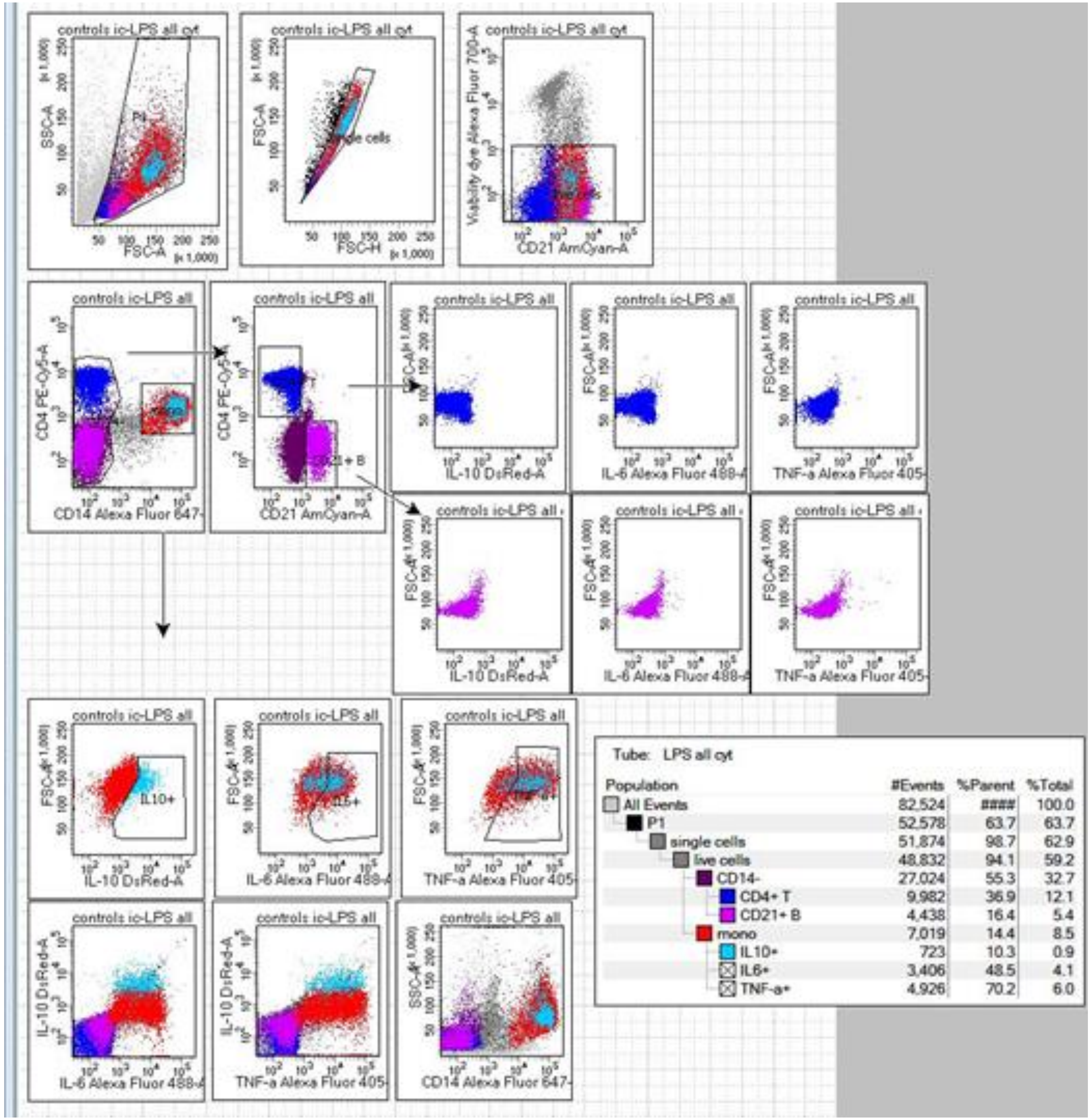

Population	#Events	%Parent	%Total
All Events	82,524	####	100.0
P1	52,578	63.7	63.7
single cells	51,874	98.7	62.9
live cells	48,832	94.1	59.2
CD14-	27,024	55.3	32.7
CD4+ T	9,982	36.9	12.1
CD21+ B	4,438	16.4	5.4
mono	7,019	14.4	8.5
IL10+	723	10.3	0.9
IL6+	3,406	48.5	4.1
TNF-a+	4,926	70.2	6.0

Figure S1

C, LPS,

Idiotypic antibody

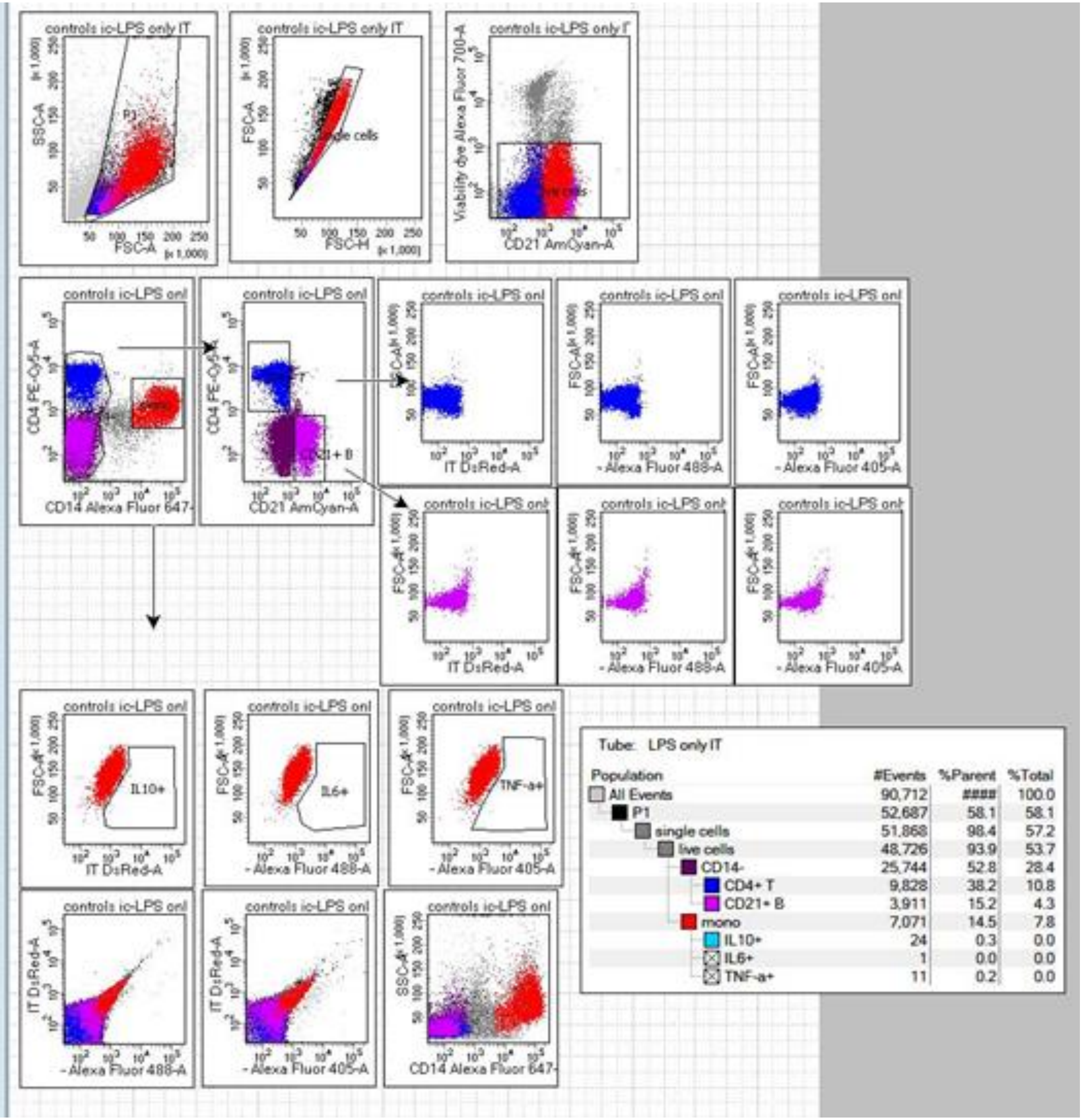

Figure S1

D, wild-type 293T cells

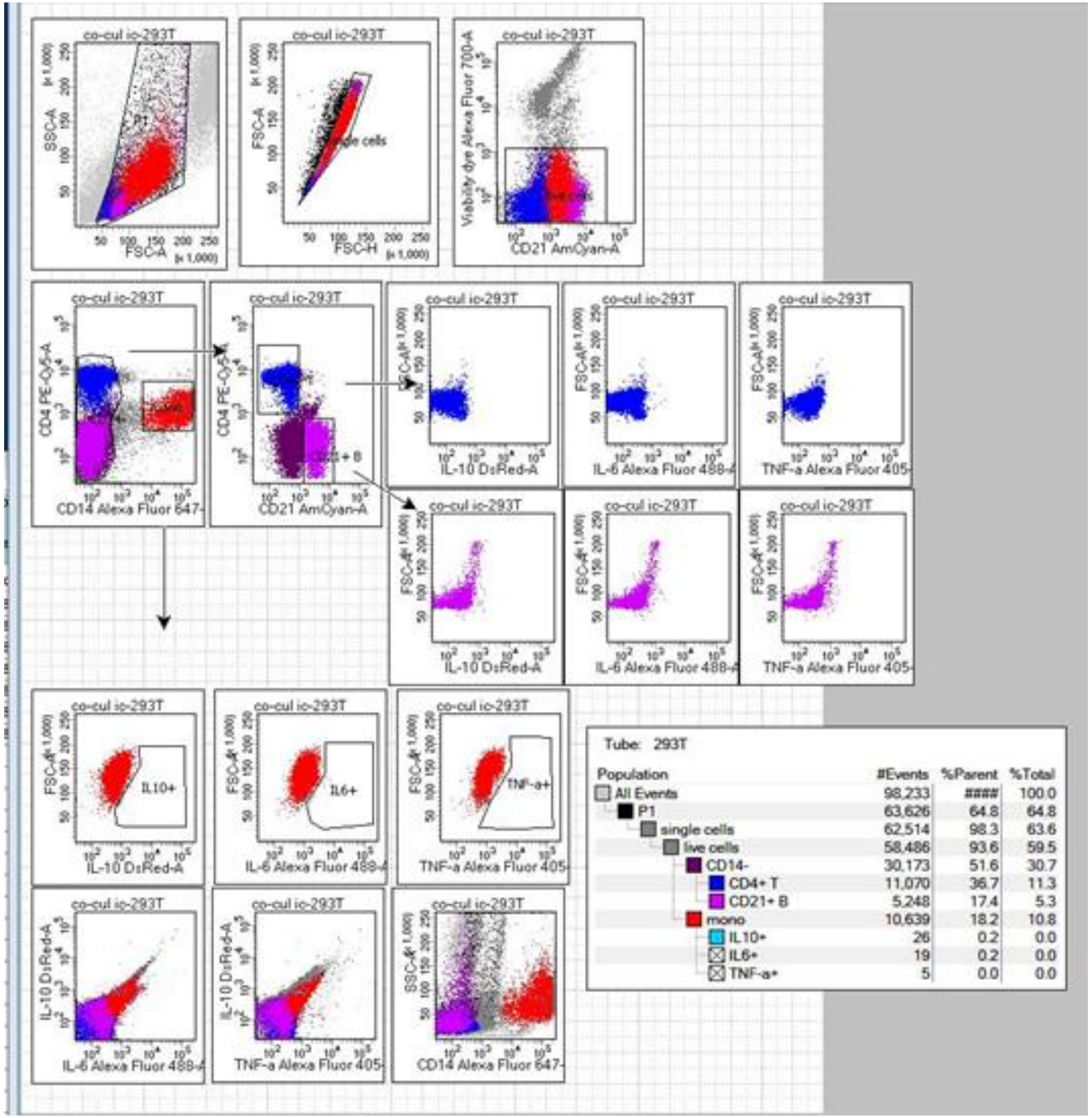

Population	#Events	%Parent	%Total
All Events	98,233	####	100.0
P1	63,626	64.8	64.8
single cells	62,514	98.3	63.6
live cells	58,486	93.6	59.5
CD14-	30,173	51.6	30.7
CD4+ T	11,070	36.7	11.3
CD21+ B	5,248	17.4	5.3
mono	10,639	18.2	10.8
IL10+	26	0.2	0.0
IL6+	19	0.2	0.0
TNF-a+	5	0.0	0.0

Figure S1

E, p15E-expressing 293T cells

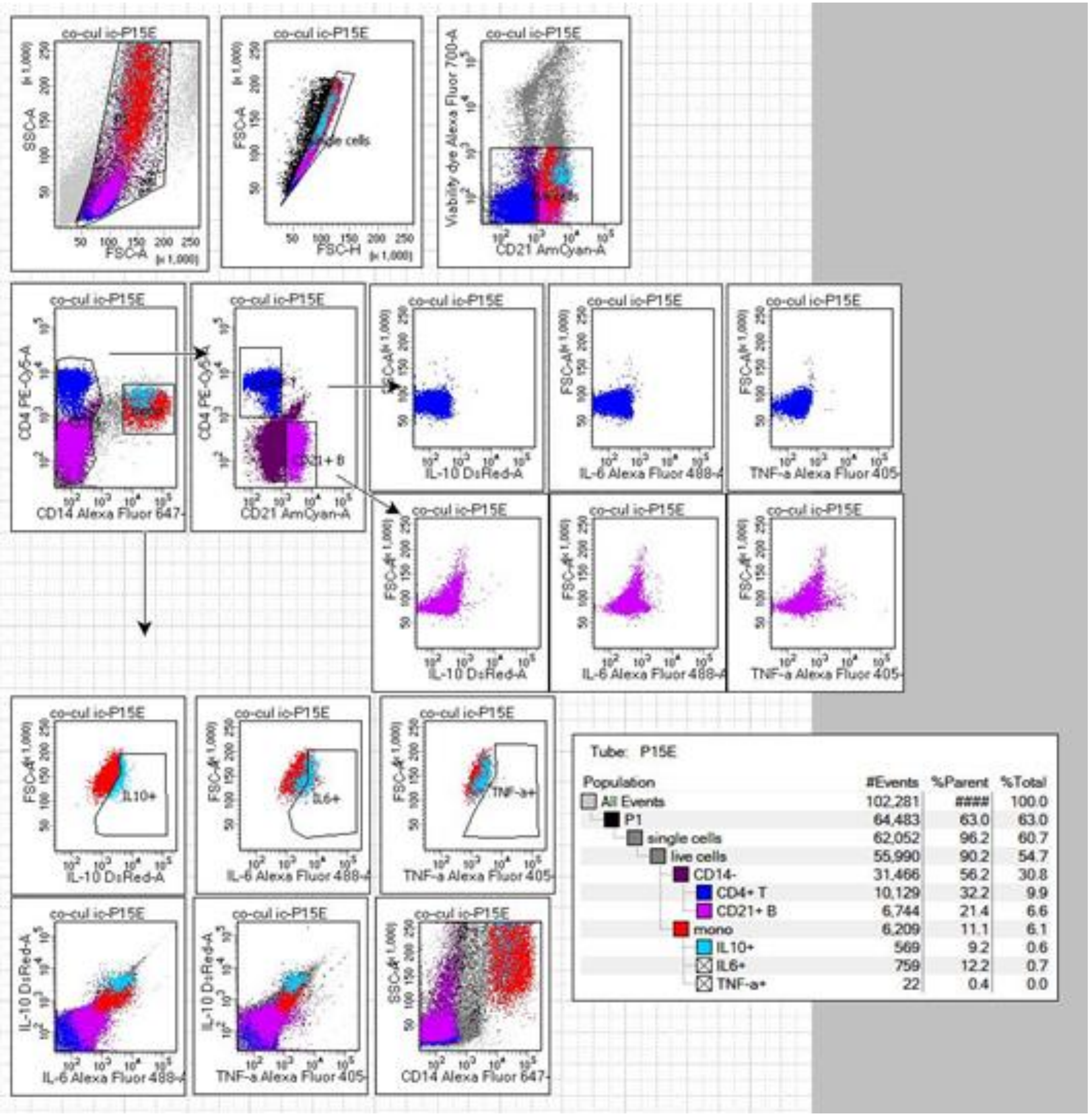

Population	#Events	%Parent	%Total
All Events	102,281	####	100.0
P1	64,483	63.0	63.0
single cells	62,052	96.2	60.7
live cells	55,990	90.2	54.7
CD14-	31,466	56.2	30.8
CD4+ T	10,129	32.2	9.9
CD21+ B	6,744	21.4	6.6
mono	6,209	11.1	6.1
IL10+	569	9.2	0.6
IL6+	759	12.2	0.7
TNF-a+	22	0.4	0.0

Figure S1

F, PERV-producing 293T cells

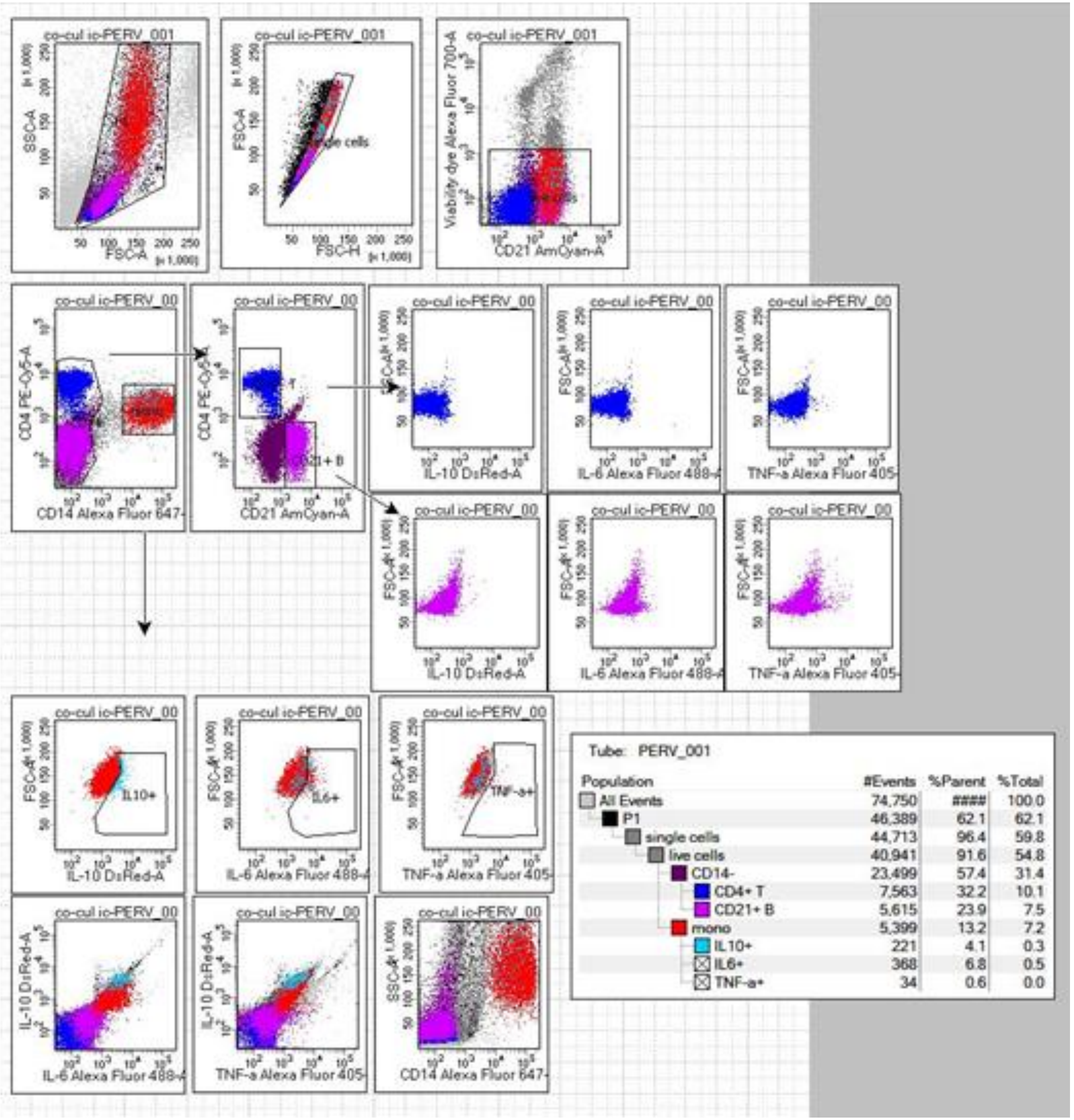

Figure S2

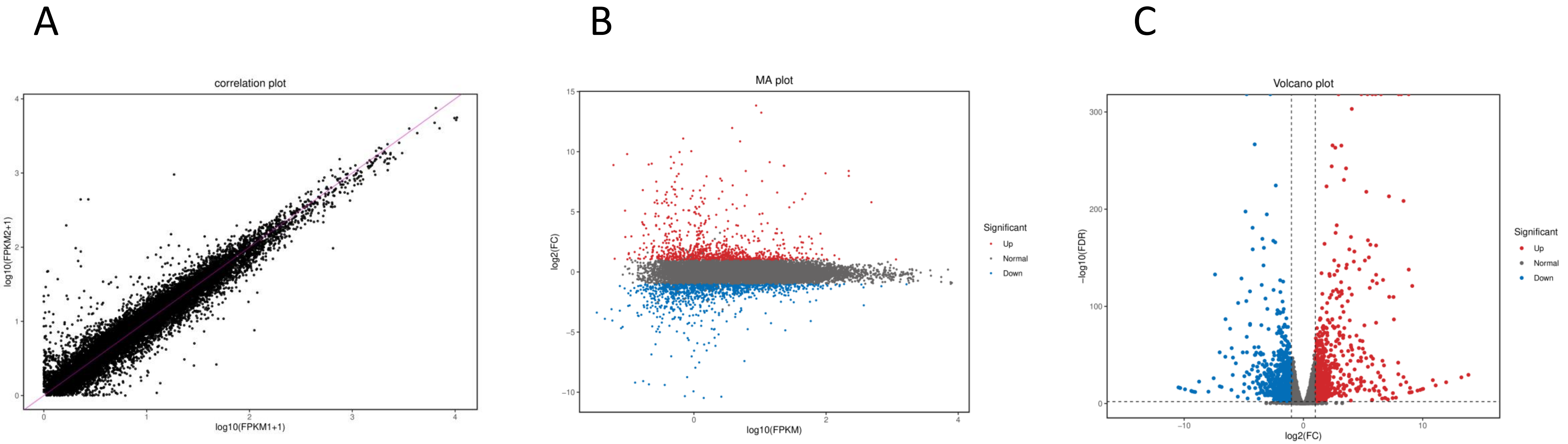
A
B
C
correlation plot
log10(FPKM2+1)
log10(FPKM1+1)
MA plot
log2(FC)
log10(FPKM)
Significant
Up
Normal
Down
Volcano plot
−log10(FDR)
log2(FC)
Significant
Up
Normal
Down
